# Feeder-cell-free system for *ex vivo* production of natural killer cells from cord blood hematopoietic stem and progenitor cells

**DOI:** 10.3389/fimmu.2025.1531736

**Published:** 2025-02-20

**Authors:** Marta Martin Corredera, Juliette Paillet, Pierre Gaudeaux, Tifanie Blein, Hanem Sadek, Pauline Rault, Asma Berriche, Jeanne Roche-Naude, Chantal Lagresle-Peyrou, Tayebeh-Shabi Soheili, Isabelle André, Ranjita Devi Moirangthem, Olivier Negre

**Affiliations:** ^1^ Smart Immune, Research & Development department, Paris, France; ^2^ Laboratory of Human Lymphohematopoieisis, Imagine Institute, INSERM UMR 1163, Université Paris Cité, Paris, France; ^3^ Biotherapy Clinical Investigation Center, Groupe Hospitalier Universitaire Ouest, AP-HP, INSERM, Paris, France

**Keywords:** natural killer (NK), cord blood (CB), hematopoietic stem and progenitor cells (HSPCs), feeder-cell-free, cytotoxicity, immunotherapy

## Abstract

**Introduction:**

Natural Killer (NK) cells hold significant promise as therapeutic agents in immuno-oncology due to their ability to target and eliminate cancerous and infected cells without causing graft-versus-host disease or cytokine release syndrome. However, the limited availability of robust, scalable methods for generating clinical-grade NK cells remains a limiting factor to broader clinical application.

**Methods:**

Here we report the development of a novel feeder-cell-free culture system optimized for producing NK cells from cord blood-derived CD34^+^ hematopoietic stem and progenitor cells (HSPCs). Our method eliminates the need for feeder cells while achieving high yields of NK cells that exhibit unique marker expression and cytotoxic functions. Cord blood CD34^+^ HSPCs were cultured in our established hDLL 4 culture system and generated large numbers of human T lymphoid progenitors (ProTcells) in 7 days. ProTcells were further cultured in a hDLL4-free, feeder-cell-free system for NK cell differentiation and supplemented with cytokines. Following a 7- or 14-day culture, this method produced highly pure NK cell populations (>90% CD3^–^CD56^+^).

**Results:**

Flow and mass cytometric analysis confirmed the expression of activating receptors, transcription factors (ID2, T-bet) and cytotoxic molecules (perforin, granzyme A/B), all essential for ProT-NK cell functionality. These cells are in an immature state, indicated by the absence of maturation markers (CD16, KIRs). Functional assays demonstrated that these ProT-NK cells are capable of degranulation and cytokines production (TNFα) upon stimulation with K562 target cells and showed cytotoxicity against K562 cells superior to that of Peripheral Blood (PB)-NK. In NSG-Tg(hIL-15) mice, ProT-NK cells colonize bone marrow, the liver, and the spleen and persist and mature in bone marrow for at least 9 days post-injection. Compared to ProT-NK D21, ProT-NK D14 was superior in functional and homing potential. *In vivo*, an anti-tumor assay that uses a subcutaneous K562 model has demonstrated the anti-tumor potential of ProT-NK cells.

**Discussion:**

Our ex vivo culture process supports scalable ProT-NK cell production in high yields, reducing dependency on feeder cells and mitigating contamination risks. Our findings demonstrate the feasibility of generating large, functional NK cell populations from HSPCs isolated from readily available cord blood sources and offer an efficient alternative to PB-NK cell therapies.

## 1 Introduction

Natural killer (NK) cells are cytotoxic innate lymphoid cells that play a crucial role in defending against infections and malignancies as they can rapidly kill virus-infected and tumor cells independently of major histocompatibility complex (MHC) restriction (1–5). Human NK cells are identified as CD56^+^CD3^-^ cells without T cell receptor (TCR) expression. Their activation and consequent target cell killing are tightly regulated by a balance of activating and inhibitory signals triggered when a variety of germline-encoded activation receptors, such as DNAX accessory molecule-1 (DNAM-1), natural cytotoxicity receptors (NCRs), C-type lectin receptors natural killer group 2D (NKG2D), natural killer group 2C (NKG2C) and activating killer immunoglobulin-like receptors (KIRs), along with inhibitory KIRs and the natural killer group 2A (NKG2A)/CD94 heterodimer are bound to their specific ligands on target cells (6). The inhibitory KIRs play a crucial role in preventing healthy self-cells from NK cell-mediated killing by binding to human leukocyte antigen (HLA) class 1 and/or HLA-E molecules expressed on healthy cells (7). In the process of haploidentical allogeneic stem cell transplantation, inhibitory KIRs of donor NK cells cannot engage recipient HLA class I molecules (missing-self) a phenomenon that activates donor NK cells that contribute to the curative graft-versus-tumor (GVT) effect (3, 8, 9). NK cells employ various mechanisms to kill their target cells: via targeted release of cytotoxic molecules (perforin and granzymes); by initiating death receptor-mediated apoptosis through Fas-L, tumor necrosis factor (TNF)-related apoptosis-inducing ligand (TRAIL) and membrane TNF-α (mTNF-α); or by antibody dependent cell-mediated cytotoxicity (ADCC) (1, 10–12).

Unlike T cells, NK cells need no prior antigen sensitization and clonal expansion to kill tumor cells rapidly, so NK cells have gained significant attention in the field of cancer immunotherapy (13). Several clinical studies have demonstrated the safety of infusing allogeneic NK cells without causing, as T cells can, graft-versus-host disease (GVHD), neurotoxicity and cytokine release syndrome (CRS); these studies have had promising results (3, 4, 14–17) and prompted efforts to develop “off-the-shelf” NK cell products for cancer immunotherapies. Multiple clinical trials ongoing or planned use allogeneic NK cells (non-modified or genetically modified) alone or in combination with monoclonal antibodies to assess the safety and effectiveness in treating several cancers, including those in advanced solid tumors (18, 19).

In most cases, peripheral blood (PB)-NK, cord blood (CB)-NK and NK92 (an NK cell line) cells are used as sources for therapeutic NK cells. However, the relative scarcity of PB-NK and CB-NK cells in PB (5-15% of PB lymphocytes) and CB (15-30% of CB lymphocytes) (20, 21) requires their ex vivo expansion before clinical use to produce enough NK cells. In addition, the clinical benefits often last only a few months due to low NK cell persistence, which typically ranges from a few days to 4 months, with an average of 7 days, so high cell doses with multiple injections are needed to reach clinical efficacy (22). Currently, irradiated feeder cells such as genetically modified K562 (a chronic myelogenous leukemia cell line) expressing membrane-bound IL-15 or IL-21 are used for clinical-grade expansion of PB-NK or CB-NK cells. Yet these expansion processes may pose regulatory challenges and are associated with high donor inter-variability that yields varying treatment outcomes. Additionally, PB-NK and CB-NK cells are known to be difficult to transduce. The required irradiation of NK92 cells before infusion prevents their *in vivo* proliferation and long-term efficacy (23, 24). Current clinical studies show that insufficient NK cell expansion methods pose the main obstacle to NK cell immunotherapy.

Alternatively, ex vivo differentiation of stem cells from different sources has been used to generate NK cells, including hematopoietic stem and progenitor cells (HSPCs) isolated from bone marrow, mobilized PB (mPB) or CB, human embryonic stem cells (hESCs) and induced pluripotent stem cells (iPSCs) (25–40). Of these, CB has emerged as the preferred source of CD34^+^ cells, due to its primitive stem cell characteristics and high proliferative capacity. Several groups have reported efficient generation of CB-CD34^+^-derived NK cell products that display enhanced effector functions and cytolytic activity against tumor cells *in vitro* and *in vivo* (26–33). However, most of these NK cell generation methods involve long culture processes ranging from 28 to 100 days; many of them use feeder cells. Given the existing challenges of expanding and producing clinical-grade NK cells, efforts are being made to develop efficient strategies for producing immunotherapeutic NK cells to facilitate a wide application of NK cell immunotherapy.

We had previously established a culture system without feeder cells that uses immobilized human delta-like ligand 4 (hDLL4) to produce large numbers of unmodified or genetically modified T cell progenitors (ProTcells) from CB or mPB HSPCs (41). These ProTcells were shown to efficiently differentiate into T cells both *in vitro* and *in vivo* (41). In this study, we investigated whether ProTcells were still able to differentiate into other lymphoid cell lineages beyond T cells. Unexpectedly, we found that ProTcells generated in our hDLL4-based system could efficiently and rapidly differentiate into NK cells (CD3^-^CD56^+^) under specific NK cell differentiation conditions. This led to the establishment of an efficient and scalable feeder-cell-free method for producing a pure population of functional NK cells in large numbers from CB CD34^+^ cells. We show that robust numbers of NK cells can be generated from CB CD34^+^ cells during a short period of 14 days in a two-step feeder cell-free culture process: in the first 7 days hDLL4 culture is used to produce lymphoid progenitors; in the second 7 days those progenitors differentiate into NK cells. Although the ProT-NK cells generated exhibit an immature phenotype, they express NK cell transcription factors, activation receptors, chemokine receptors and cytotoxic molecules (perforin and granzyme B), and produce TNF-α in response to K562 cells and can kill K562 cells both *in vitro* and *in vivo* in NSG-hIL15 transgenic mice. Finally, we showed that our NK cell production method can be combined with transduction to generate chimeric antigen receptor (CAR) NK cells. In sum, our novel method for NK cell generation may provide a promising platform for producing large quantities of non-modified or genetically modified NK cells for clinical use and contribute to broader and more effective application of NK cell-based adoptive immunotherapy for cancer and viral infections.

## 2 Materials and methods

### 2.1 Human samples

Umbilical cord blood (CB) samples were collected from donors at AP-HP Hôpital Saint-Louis (Unité de Thérapie Cellulaire, CRB-Banque de Sang de Cordon, Paris, France) after written informed consent and in compliance with ethical guidelines. Enriched CB CD34+ cells for specific experiments were purchased from Lymphobank (Besançon, France). Peripheral blood from healthy adult donors was obtained with written informed consent from the French Blood Transfusion Institute (Etablissement Français du Sang) (Paris, France).

### 2.2 Selection of CD34^+^ hematopoietic stem and progenitor cells

Cord blood mononuclear cells (MNCs) were isolated by density gradient centrifugation using Lymphocyte Separation Medium (LSM) (Eurobio Scientific, Les Ulis, France). CD34^+^ cells were then enriched immunomagnetically from MNCs using Indirect human CD34^+^ cell isolation kit (Miltenyi Biotech, Bergisch Gladbach, Germany) following manufacturers’ instructions. The purity of isolated CD34^+^ cells exceeded 90%.

### 2.3 Isolation of Peripheral blood NK cells

Peripheral blood mononuclear cells (PBMCs) were isolated from adult healthy donor peripheral blood using density gradient centrifugation with LSM. PB-NK cells were then immunoselected negatively from PBMCs in a magnetic separator using NK cell isolation kit (Miltenyi Biotech, Bergisch Gladbach, Germany) according to manufacturers’ instructions. The purity of isolated CD3^-^CD56^+^ cells exceeded 90%.

### 2.4 Cell lines

K562 myelogenous leukemia cell line was purchased from ATCC (Manassas, VA, USA). K562-GFP/Luc cell line was purchased from Creative Biogene (CSC-RR0374, New York, NY, USA). Both cell lines were cultured in Roswell Park Memorial Institute 1640 Medium, with GlutaMAX™ Supplement (RPMI) (Gibco, Courtaboeuf, France) containing 50 µg/ml gentamycin and 10% Fetal Bovine Serum (FBS Gibco) (Gibco, Courtaboeuf, France). Puromycin (0.8 µg/mL) (InvivoGen, Toulouse, France) was added for the selection of stably transduced K562-GFP/Luc cells. They were passaged every 2-3 days.

### 2.5 Two-step ex-vivo generation of ProT-NK cells

#### 2.5.1 hDLL4 culture step: Culture of CD34^+^ HSPCs on immobilized Fc-hDLL4

Human CD34^+^ HSPCs isolated from CB were cultured for 7 days at 5 x 10^4^ cells/mL in culture plates/flasks coated with Fc-hDLL4 fusion protein (6.4 μg/mL, Merck-Millipore, SIFP3 S25-055-CM, Bordeaux, France) and RetroNectin^®^ (25 μg/ml, Takara Bio Europe, Saint-Germain-en-Laye, France) in Minimum Essential Medium α (α-MEM) (Gibco, Courtaboeuf, France) supplemented with 20% defined FBS (FBS HyClone) (HyClone, Cytiva Europe GmbH, Velizy-Villacoublay, France), 50 µg/mL gentamycin (Gibco, Courtaboeuf, France) and human cytokines: 100 ng/mL interleukin 7 (hIL-7), 100 ng/mL FMS-like tyrosine kinase 3 ligand (hFlt3-L), 100 ng/mL stem cell factor (hSCF), 100 ng/mL thrombopoietin (hTPO) (all from PeproTech, Neuilly-sur-Seine, France) and 100 ng/mL tumor necrosis factor alpha (TNFα) (R&D Systems, Minneapolis, MN, USA) to obtain lymphoid progenitors (ProTcells).

#### 2.5.2 Ex vivo NK cell differentiation and expansion

NK cell differentiation and expansion were performed in a feeder cell-free culture system. The lymphoid progenitors (ProTcell) obtained after 7 days (96- to 24-well plates for small-scale and T25 or T75 for large-scale cultures) of Fc-hDLL4 culture were seeded at 6 x 10^4^ cells/mL and cultured for either 7 (hereafter referred as ProT-NK D14) or 14 days (hereafter referred as ProT-NK D21) in up to culture 6 well plates (for small-scale culture) or T150 culture flasks (for large-scale culture) in RPMI supplemented with 10% defined FBS HyClone, 50 µg/ml gentamycin (Gibco, Courtaboeuf, France) and human cytokines: 20 ng/mL hIL-7, 50 ng/mL hFlt3-L, 50 ng/mL hSCF, 20 ng/mL interleukin 15 (hIL-15) (all from PeproTech, Neuilly-sur-Seine, France) and 500 IU/mL recombinant interleukin 2 (hIL-2) (Proleukin, Novartis, Liverpool, UK). Cell concentration was maintained at 1.5 x 10^6^ cells/mL from day 7 to day 14.

### 2.6 Peripheral blood NK cell expansion

Isolated PB-NK cells were cultured and expanded for 10 to 20 days in NK MACS medium (Miltenyi Biotech, Bergisch Gladbach, Germany) supplemented with 1% NK MACS Supplement, 5% human serum albumin (HSA) (BioWest, Nuaillé, France), 50 µg/ml of gentamycin (Gibco, Courtaboeuf, France) and human cytokines: 20 ng/mL hIL-15 (PeproTech, Neuilly-sur-Seine, France) and 500 IU/mL recombinant hIL-2 (Proleukin, Novartis, Liverpool, UK), following manufacturers’ instructions.

### 2.7 Transduction

CB CD34^+^ HSPCs were preactivated overnight on Fc-hDLL4 fusion protein and RetroNectin^®^-coated wells in X-vivo 20 medium (Lonza, Walkersville, MD, USA) containing the same cytokines (hSCF, hFLT3-L, hTPO, hIL-7, hTNFα) described above (hDLL4 culture step). The cells were then transduced under the preactivation condition with a lentiviral vector encoding an anti-CD19 chimeric antigen receptor (CAR) (FMC63) (19-scr, Flash Therapeutics, Toulouse, France) at a multiplicity of infection (MOI) of 100, in presence of 4 µg/ml protamine sulfate (Sigma-Aldrich, St. Louis, MO, USA). After 6 hours of transduction, the cells were washed with α-MEM (Gibco, Courtaboeuf, France) supplemented with 20% FBS HyClone and cultured for a total of 7 days in Fc-hDLL4 culture conditions to produce transduced lymphoid progenitors.

### 2.8 Flow cytometry

The antibodies listed in **Table 1** were used for flow cytometry (FC) analyses. For surface staining, cells were incubated with 7-AAD (Miltenyi Biotech, Bergisch Gladbach, Germany) and the appropriate antibodies for 15 to 45 min at 4°C, washed and resuspended in FACS buffer (0.5% BSA Fraction V (EuroBio Scientific, Les Ulis, France) and 2mM EDTA (ThermoFisher Scientific, Dardilly, France) in 1X PBS (Eurobio Scientific, Les Ulis, France). For intracellular stainings, cells were first surface stained with Viobility™ Fixable Dye (Miltenyi Biotech, Bergisch Gladbach, Germany) and the appropriate antibodies for 30min in ice and washed with FACS buffer. The surface-stained cells were fixed and permeabilized using either BD Cytofix/Cytoperm Fixation/Permeabilization Kit (BD Biosciences, Belgium) or eBioscience Foxp3/Transcription Factor Staining Buffer Set (Thermo Fisher Scientific, Dardilly, France) according to manufacturer’s instructions and stained with the appropriate antibodies for 30min in ice, and then washed and resuspended in FACS buffer. Data acquisition was performed using Novocyte^®^ flow cytometer (Agilent Technologies, Santa Clara, CA, USA), and the acquired data were analyzed using Kaluza Analysis Software (version 2.1, Beckman Coulter, Krefeld, Germany). All gatings were performed on live cells which were determined by exclusion of 7-AAD or Viobility dyes.

**Table 1:** Antibody panels used for flow cytometry.

Antibody	Fluorochrome	Clone	Supplier	Reference
hCD3	BV711	SK7	Biolegend	344838
hCD7	BV510	M-T701	BD Pharmingen	563650
hCD16	BV510	3G8	Biolegend	302048
hCD34	BV786	581	BD Biosciences	743534
hCD45	BV421	HI30	Biolegend	304032
hCD56	APC-Vio770	REA196	Miltenyi	130-114-548
hCD94	FITC	DX22	Biolegend	305504
hCD161	FITC	191B8	Miltenyi	130-113-592
hNKp30	APC	P30-15	Biolegend	325228
hNKp44	BV650	P44-8	BD Biosciences	744302
hNKp46	PE	9E2/Nkp46	BD Pharmigen	557991
hDNAM-1	BV421	REA1040	Miltenyi	130-123-522
hNKG2C	APC	REA797	Miltenyi	130-117-398
hNKG2D	APC	REA797	Miltenyi	130-111-846
hNKG2A	PE-Vio770	REA110	Miltenyi	130-113-567
hKLRG1	PE	REA261	Miltenyi	130-120-566
hKIR2DL2/3	FITC	DX27	BD Pharmigen	559784
hKIR3DL1/2	PE-Vio770	REA970	Miltenyi	130-116-180
hPerforin	PE	REA1061	Miltenyi	130-118-117
Granzyme B	APC	REA226	Miltenyi	130-120-703
CD107a	PE-Vio770	REA792	Miltenyi	130-111-622
hTNFα	PE	REA656	Miltenyi	130-118-974
hIFNγ	APC	45-15	Miltenyi	130-113-490
hEOMES	PE	WD1928	eBioscience	12-4877-42
hT-bet	APC	REA102	Miltenyi	130-119-783
hID-2	PE-Cy7	ILCID2	eBioscience	25-9475-82
mTER119	BV650	TER-119	Biolegend	116235

### 2.9 Degranulation and cytokine induction assay

ProT-NK D14, ProT-NK D21 or expanded PB-NK cells were stimulated with K562 cells by co-incubating them at a ratio of 1:2 (NK to K562) for 6 hours in RPMI supplemented with 10% FBS HyClone inside an incubator at 37°C, 5% CO_2_. CD107a antibody (Miltenyi Biotech, Bergisch Gladbach, Germany) was added at the beginning of co-incubation. After 1 hour of incubation, protein transport inhibitors BD GolgiPlug and GolgiStop (BD Biosciences, Belgium) were added and further incubated for 5 hours. Cells were analyzed by FC after a total of 6 hours of co-incubation.

### 2.10 Cytotoxicity assay

FC-based cytotoxicity assay was performed using K562 cells as target cells. Target cells were, labelled with CellTrace Violet (CTV) dye (Invitrogen, ThermoFisher Scientific, Dardilly, France) according to manufacturer’s instructions, to distinguish them (CTV+) from effector NK cells (CTV-). Labelled target cells were incubated with effector NK cells (ProT-NK D14, ProT-NK D21 or expanded PB-NK) at different effector-to-target ratios in RPMI supplemented with 10% FBS HyClone and 100 IU/mL of recombinant hIL-2 (Proleukin, Novartis, Liverpool, UK) for 5 hours at 37°C, 5% CO_2_ inside an incubator. Cells were then stained with 7-AAD and analyzed by FC. The specific killings by effector NK cells were calculated as:


$$\frac{{\left[\%7\text{AAD}+\text{CTV}+\;\text{cells}\right]}_{\left(\text{Effector}+\text{Target}\right)\text{well}}-{\left[\%7\text{AAD}+\text{CTV}+\;\text{cells}\right]}_{\left(\text{Target}\;\text{only}\right)\text{well}}\;}{{\left[\%7\text{AAD}+\text{CTV}+\;\text{cells}\right]}_{\left(\text{Target}+\text{Detergent}\right)\text{well}}-{\left[\%7\text{AAD}+\text{CTV}+\;\text{cells}\right]}_{\left(\text{Target}\;\text{only}\right)\text{well}}}\;\times \;100$$

### 2.11 Mass cytometry

Mass cytometry analysis was performed using specifically designed antibody panels for phenotypic (**Supplementary Table S1**) and functional analyses (**Supplementary Table S2**). Metal isotope labelled antibodies were purchased from Fluidigm (San Francisco, CA, USA). Antibodies listed in **Supplementary Table S3** were labelled manually using Maxpar X8 Antibody Labelling Kit for Lanthanides and Maxpar MCP9 Antibody Labelling Kit for Cadmium (Fluidigm, San Francisco, CA, USA), following manufacturer’s instructions. For surface staining, cells were incubated with Cisplatine Cell-IDTM (Fluidigm, San Francisco, CA, USA) at 2.5μM for 5 minutes at room temperature (RT) to label dead cells. and the appropriate antibodies (**Supplementary Tables S1, S2**) for 30 min at 4°C, washed and resuspended in FACS buffer and fixed with 1 mL of staining solution containing 1.6% paraformaldehyde (Sigma-Aldrich, St. Louis, MO, USA) for 10 minutes at 4°C. For intracellular stainings, cells were first incubated with Cisplatine Cell-IDTM (Fluidigm, San Francisco, CA, USA) as described above and surface-stained with the appropriate antibodies for 30 min in ice and washed with FACS buffer. The surface-stained cells were fixed and permeabilized using Foxp3/Transcription Factor Staining Buffer Kit (Tonbo Biosciences, San Diego, CA, USA) and stained with the appropriate intracellular antibodies for 1 hr at 4°C, and then washed and resuspended in FACS buffer. Following extracellular or intracellular staining, the cells were washed and resuspended in 1mL of Fix and Perm Buffer containing 1:4000 of Iridium intercalator (Fluidigm, San Francisco, CA, USA) and incubated overnight at 4°C. The stained cells were stored at -80°C until analysis. The data was acquired on Helios mass cytometer with CyTOF software version 6.7.1014 (Fluidigm, San Francisco, CA, USA) at the Cytometry Platform of La Pitié-Salpétrière Hospital. Mass cytometry standard files were normalized using CyTOF Software v. 6.7.1014. To identify cell clusters from CyTOF data, either Uniform Manifold Approximation and Projection [UMAP (42)] or cluster identification [FlowSOM (43)] were run. For both analyses, samples were run on equal numbers of events per sample. To visualize each cell on a two-dimensional map, a UMAP algorithm was applied with the following setting: 200 epochs, minimum distance of 0.4, learning rate of 1, number of nearest neighbors of 15. Clustered heatmap used Euclidian distance and standard normalization in Omiq. To automatically identify differing cell clusters among groups, FlowSOM algorithm was run with 7 nearest neighbors (k = 7). The distinct clusters were manually annotated based on the selected markers: CD56, CD161, CD94, NKG2D, CD57, CD16, KIRs, NCRs, and CXCR4.

### 2.12 Mice

NOD.Cg-Prkdcscid Il2rgtm1Wjl Tg(IL-15)1Sz/SzJ (NSG-Tg(hIL-15) mice were purchased from Jackson Laboratories and bred in-house at LEAT Institut Necker Enfants Malades animal facility (registration number A75-15-34). The studies conducted adhered to French ethical regulations and all the procedures performed were in accordance with protocols approved by the “Services Vétérinaires de la Préfecture de Police de Paris” (Veterinary Services of the Paris Police Department) and the “Comité d’Ethique en matière d’Expérimentation Animale Paris Descartes” (Paris Descartes Animal Experimentation Ethics Committee) (CEEA-034) under the number APAFIS#38814- 202202011338747_v8, Université Paris Descartes, Paris, France.

### 2.13 *In vivo* homing, persistence and maturation studies

NSG-Tg(hIL-15) mice (8 -12 weeks old) were conditioned with a low dose of busulfan (30 mg/kg), administered intraperitoneally in 2 split doses of 15 mg/kg in two consecutive days at an interval of 24h to improve human cell engraftment. One day post-conditioning, mice were anesthetized with isoflurane (Vetflurane, Virbac, Carros, France), and injected intravenously with 20 x 10^6^ of either PB-NK, ProT-NK D14 or ProT-NK D21 cells through retro-orbital plexus. Bone marrow, liver, spleen and blood were analyzed using FC 5 and 9 days post-injection to assess NK cell engraftment.

### 2.14 *In vivo* anti-tumor study

To assess the *in vivo* anti-tumor activity of ProT-NK cells, human acute myeloid leukemia (AML) tumor xenograft mouse model was established in NSG-Tg(hIL-15) mice (8 -12 weeks old). Mice were conditioned and anesthetized as described in the previous section. One day after conditioning, they were injected subcutaneously with K562-Luc cells either alone (0.5 x 10^6^ cells/mouse) or mixed with PB-NK or ProT-NK D14 cells (20 x 10^6^ cells/mouse) under the skin overlying the lower back. Tumor progression was monitored via bioluminescent imaging (BLI) every 3-4 days for 50 days using IVIS Spectrum CT (Revvity, Waltham, MA, USA). D-luciferin (150 mg/kg, Revvity, Waltham, MA, USA) was intraperitoneally injected, and images were captured under isoflurane anesthesia within 20 min of injection. Data were analyzed using Living Image software (IVIS Imaging Systems), with BLI and X-ray superimposition signals quantified after 3D tomography. A region of interest was determined by delineating the bioluminescent zone, and the absolute number of cells was obtained using *in vitro* calibration with corresponding K562-GFP/Luc cells. Bioluminescence data is expressed as photons/s. Mice were sacrificed by cervical dislocation when tumors reached 1.7 cm in diameter or if specific criteria were met (e.g. severe weight loss, poor coat and skin condition, static activity or paraplegia).

### 2.15 Cell preparation from mouse organs

Mice blood samples were collected in EDTA tubes by orbital plexus bleeding with capillary tubes from anesthetized mice. The mice were then sacrificed by cervical dislocation and right femurs, spleen and liver were collected and processed for FC analysis. Bone marrow cells were flushed out of femur bones with 25G needles and 10mL syringes in RPMI supplemented with 10% FBS Gibco and filtered through 40 µM cell strainers. Spleens were mechanically dissociated using 1ml syringe plungers in RPMI supplemented with 10% FBS Gibco and passed through 40 µM cell strainers. Livers were dissociated mechanically by crushing between two glass slides for mild dissociation. The liver cell suspensions were collected and filtered through 70 µM cell strainers. Hepatocytes were separated from liver cell suspensions with low-speed centrifugation (50 rcf) and discarded. Lymphocytes were then isolated from the remaining cell suspension by density gradient centrifugation using 33% Percoll solution (OptiPrep, Stemcell Technologies, Vancouver, Canada) and resuspended in RPMI supplemented with 10% FBS Gibco.

### 2.16 Statistical analysis

Data are presented as mean ± SD for frequency variables and mean ± SEM for numerical variables. Statistical analyses were conducted using GraphPad Prism version 10. Statistical differences between groups for *in vitro* studies were evaluated using either one-way or two-way ANOVA. For *in vivo* study results, non-parametric tests were performed using (Kruskal-Wallis Test). Kaplan Meier plots were used to calculate survival probabilities and P-values were computed using log-rank (Mantel-Cox) test. Linear regression with the Wald test was used for CD161 expression and NK cell purity correlation. P-values ≤0.05 were considered as statistically significant: *p ≤ 0.05; **p ≤ 0.01; ***p ≤ 0.001, ****p ≤ 0.0001. ns: non-significant.

## 3 Results

### 3.1 Scalable and robust ex vivo production of CB-HSPC derived NK cells

**Figure 1:**
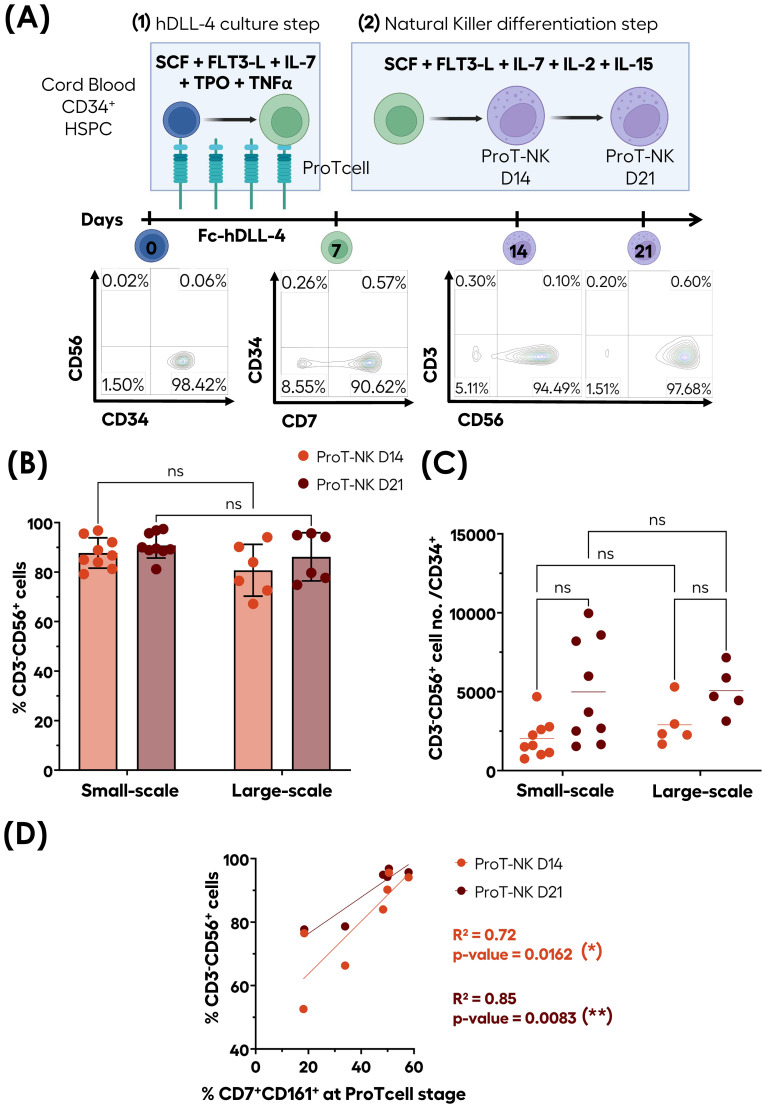
Efficient ex vivo generation of highly pure CD3^-^CD56^+^ ProT-NK cells from CB CD34^+^ cells. CD34^+^ cells isolated from CB (blue cells) were differentiated and expanded into ProTcells (green cells) using an Fc-hDLL4 immobilized ligand in presence of a cytokine cocktail for seven days. Subsequently, ProTcells (green cells) were differentiated into NK cells (violet cells) under feeder-cell-free culture conditions with a second cytokine cocktail for either 7 or 14 days. The presence of NK cells (CD3^-^CD56^+^) and their numbers were analyzed after a total of 14 or 21 days of cultures using flow cytometry (FC). **(A)** Schematic representation of ProT-NK differentiation and expansion culture protocol and representative FC plots of generated CD3-CD56+ populations. Created with Biorender.com. **(B)** and cell number per CD34^+^ cells **(C)** of CD3^-^CD56^+^ cells after 14 (ProT-NK D14: red) and 21 days (ProT-NK D21: brown) in small-scale cultures (up to 6 well-plates; mean ± SD, ProT-NK N=9) and large-scale cultures (T150 flasks; mean ± SD, ProT-NK N=6(*). The fold expansion represents the total NK cell number generated per CB CD34^+^ cell seeded. Statistical significance was assessed using two-way ANOVA: p values ≤ 0.05 were considered as significant. ns, non-significant. **(D)** Linear regression showing the correlation between CD161 expression at the ProTcell stage and CD3^-^CD56^+^ cell frequency post-NK differentiation (ProT-NK D14 N=7, ProT-NK D21 N=6). The statistical significances were determined by the Wald test for linear regression: *p ≤ 0.05; **p ≤ 0.01. N represents the number of donors.

We have shown that CB CD34^+^ HSPCs cultured during 7 days in a feeder-cell free system containing immobilized hDLL4 and a specific set of cytokines resulted in the production of >90% of CD7 expressing T-cell progenitors (ProTcells) that express intracellular CD3ϵ and transcription factor GATA3, known to be associated with T-cell commitment (41, 44). To exclude any potential other than T-cell differentiation, we cultured ProTcells in conditions promoting differentiation into other lymphoid lineages. Unexpectedly, we observed efficient and rapid differentiation of ProTcells into NK cells (ProT-NK) (CD3^-^CD56^+^), after culturing them in a hDLL4-free and feeder-cell-free system with a specific cytokine cocktail for NK cell differentiation for a total of 14 or 21 days. The purity of ProT-NK cell product reached an average range of 80 to 95% at both day 14 (ProT-NK D14) and day 21 (ProT-NK D21), with minimal detection of CD3^+^ cells (**Figure 1A**).

This NK cell production process had been tested on small-scale, with the first step performed in 96- to 24-well plates and the second step in up to 6-well plates. We aimed to evaluate the feasibility of scaling up this culture process and to assess whether the scale-up would affect the quality of the generated NK cells. In the large-scale process, the first step was performed in T25/T75 flasks, and the second step was carried out T150 flasks (**Figure 1B**). No significant differences were observed in NK cell (CD3^-^CD56^+^) frequencies between small-scale and large-scale production for both day 14 and day 21 ProT-NK products with their frequencies ranging from 87.7 ± 6.1% (D14) to 90.8 ± 5.1% (D21) (mean ± SD) for small-scale and from 82.4 ± 10.8% (D14) to 88.4 ± 8.9% (D21) (mean ± SD) for large-scale (**Figure 1B**), suggesting that NK cell purity was not affected by the scale-up. The analysis of NK cell yields from different CD34^+^ HSPC donors (**Figure 1C**) showed that a single CD34^+^ CB HSPC produced an average of 2037 ± 406 (D14) and 4981 ± 1087 (D21) (mean ± SEM) ProT-NK cells for small-scale and 2906 ± 633 (D14) and 5063 ± 681 (D21) ProT-NK cells for large-scale productions (**Figure 1C**). Importantly, no significant difference was observed in NK cell yields of small-scale and large-scale productions (**Figure 1C**), indicating that scale-up did not impact ProT-NK cell yield.

Since CD161 has already been reported to be highly expressed early during NK cell development (45), we investigated if ProTcells express CD161. Flow cytometry analysis revealed that a fraction of CD7^+^ ProTcells express CD161 (**Supplementary Figure S1A**). Then, we used a linear regression analysis to investigate whether there is any correlation between the percentage of CD7^+^CD161^+^ cells in ProTcell and the purity of the generated NK cell product (**Figure 1D**). The analysis revealed a strong correlation between initial proportions of CD7^+^CD161^+^ cells present at ProTcell stage and CD3^-^CD56^+^ NK cell purity in the resulting ProT-NK cell product, with an R² value of 0.72 (p = 0.0162) at day 14 and an even stronger correlation at day 21, with an R² of 0.85 (p = 0.0083) (**Figure 1D**). These findings suggest that initial percentage of CD7^+^CD161^+^ cells in ProTcell may predict NK cell purity of our ex vivo generated ProT-NK cell product and that more than 50% of CD7+CD161+ in ProTcells may be necessary to achieve over 90% of CD3^-^CD56^+^ ProT-NK cell purity. However, the CD3^-^CD56^+^ cell yield didn’t correlate with the CD7^+^CD161^+^ initial cell proportion in ProTcells (**Supplementary Figure S1B**). Collectively, these data demonstrate that our ex vivo feeder cell-free culture process reliably produces pure NK derived from CB ProTcells (ProT-NK) in large-scale without significant T cell contamination, highlighting the efficiency and scalability of our system.

### 3.2 ProT-NK cells exhibit a unique phenotype

**Figure 2:**
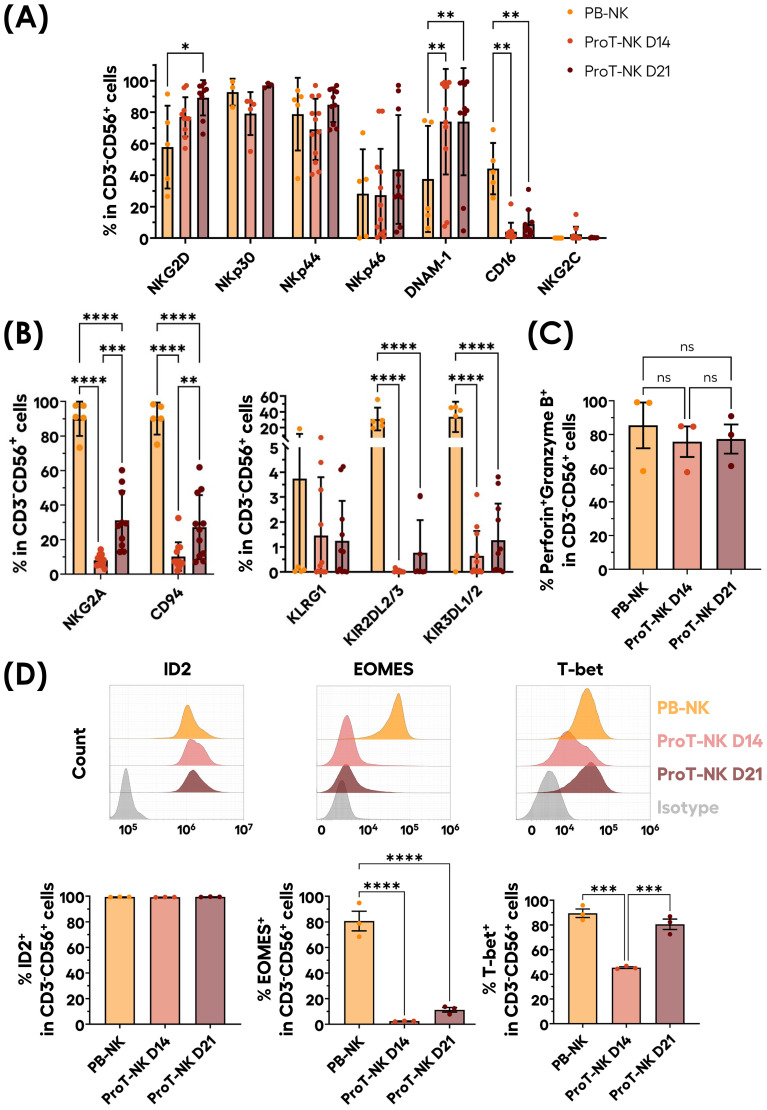
Phenotypical profile of ex-vivo generated ProT-NK cells. **(A, B)** Graphical representation of mean expression frequencies of indicated NK cell activation receptors **(A)** and inhibitory receptors **(B)** within CD3^-^CD56^+^ cells, analyzed via flow cytometry on ProT-NK cells generated after 14 (ProT-NK D14) or 21 days (ProT-NK D21) after ProT-NK expansion and differentiation culture, and day 14 expanded PB-NK cells (mean ± SD, PB-NK N=5, ProT-NK D14 N=12, ProT-NK D21 N=10 or 11). The statistical significances were calculated by two-way ANOVA: *p ≤ 0.05; **p ≤ 0.01; ****p ≤ 0.0001. **(C)** Graphs showing the mean frequency of Perforin and granzyme B co-expression among CD3^-^CD56^+^ cells in ProT-NK D14, ProT-NK D21 and day 14 expanded PB-NK cells (mean± SD, N=3). **(D)** Representative FC histograms for the expressions of NK-related transcription factors: inhibitor of DNA binding 2 (ID2) (top left panel), eomesodermin (EOMES) (top left panel), T-box expressed in T cells (T-bet) (top right panel) in ProT-NK D14, ProT-NK D21 and day 14 expanded PB-NK cells, and their corresponding mean expression frequencies (below panels) in CD3^-^CD56^+^ cells (mean± SD, N=3). The statistical significances were calculated by one-way ANOVA: ***p ≤ 0.001, ****p ≤ 0.0001. ns: non-significant. N represents the number of donors.

Next, we characterized our ProT-NK cell products and compared them with expanded PB-NK cells to assess if our ex vivo generated ProT-NK cells are similar to PB-NKs, as PB-NK cells represent standard physiologically differentiated NK cells. Flow cytometry analysis revealed that, in comparison to PB-NK cells, both ProT-NK D14 and D21 expressed similar percentages of NK cell activation receptors NKp30, NKp44, NKp46, and NKG2C (**Table 2**, **Figure 2A**, **Supplementary Figures S2, S3A**). However, ProT-NK D14 and D21 cells showed significantly higher expressions of DNAM-1 and ProT-NK D21 showed significantly higher NKG2D expression compared to PB-NK cells (**Table 2**, **Figure 2A**, **Supplementary Figures S2, S3A**). Conversely, both ProT-NK D14 and D21 display lower CD16 expressions than PB-NK (**Table 2**, **Figure 2A**, **Supplementary Figures S2, S3A**). Regarding inhibitory receptors, both ProT-NK D14 and D21 showed lower expression of KLRG1, KIR2DL2/3 and KIR3DL1/2 compared to PB-NK (**Table 3**, **Figure 2B**, **Supplementary Figure S3B**). CD94 and NKG2A were highly expressed in PB-NK, while their expressions were comparatively much lower in ProT-NK D14 and D21 (**Table 3**, **Figure 2B**, **Supplementary Figure S3B**). Overall, the ProT-NK cell phenotype is characterized by high levels of activation receptors and low levels of inhibitory markers.

**Table 2:** Mean expression frequencies of NK cell activation receptors within CD3^-^CD56^+^ cells.

	Mean % ± SD		
	PB-NK	ProT-NK D14	ProT-NK D21
NKG2D	57.76 ± 26.28	77.00 ± 12.48	89.15 ± 11.25
NKp30	92.76 ± 8.56	79.12 ± 13.64	97.07 ± 1.45
NKp44	78.78 ± 23.12	69.10 ± 19.47	84.67 ± 11.00
NKp46	28.30 ± 28.16	27.32 ± 29.40	43.61 ± 34.61
DNAM-1	37.57 ± 33.71	73.97 ± 33.51	73.96 ± 34.08
CD16	44.14 ± 16.34	4.00 ± 5.73	8.96 ± 9.17
NKG2C	0.01 ± 0.03	2.35 ± 4.81	0.21 ± 0.20

**Table 3:** Mean expression frequencies of NK cell inhibitory receptors within CD3^-^CD56^+^ cells.

	Mean % ± SD		
	PB-NK	ProT-NK D14	ProT-NK D21
KLRG1	3.74 ± 8.18	1.45 ± 2.34	1.25 ± 1.60
NKG2A	90.0 ± 9.95	8.06 ± 3.33	31.13 ± 17.02
CD94	90.09 ± 9.26	10.23 ± 8.23	27.18 ± 18.60
KIR2DL2/3	30.81 ± 14.42	0.03 ± 0.05	0.77 ± 1.31
KIR3DL1/2	33.57 ± 19.31	0.64 ± 0.99	1.27 ± 1.47

We then analyzed the expression of key cytotoxic molecules, perforin and granzyme B, which are critical for NK cell cytotoxicity (46) (**Figure 2C**, **Supplementary Figure S3C**). Both ProT-NK D14 and D21 cells showed high perforin and granzyme B expressions, comparable to expanded PB-NK cells (PB-NK: 85.4 ± 23.5%, ProT-NK D14: 75.7 ± 15.7%, ProT-NK D21: 77.3 ± 14.9%).

NK ontogeny relies on the expression of transcription factors ID2, EOMES and T-bet (47, 48). Therefore, to confirm NK identity, the expression of these transcription factors was assessed in ProT-NK cells and compared to expanded PB-NK cells (**Figure 2D**). ProT-NK cells expressed significantly lower EOMES than PB-NK (PB-NK: 80.7 ± 13.3%, ProT-NK D14: 2 ± 0.3%, ProT-NK D21: 11 ± 3%). T-bet expression was comparable between PB-NK and ProT-NK D21 (PB-NK: 89 ± 6%, ProT-NK D21: 81 ± 7%) but was lower in ProT-NK D14 cells (45 ± 1%). ID2 was highly expressed in both ProT-NK D14 and D21 cells, comparable to PB-NK (PB-NK: 99 ± 0.1%, ProT-NK D14: 99 ± 0.1%, ProT-NK D21: 99 ± 0.2%) (**Figure 2D**). In summary, ProT-NK D14 are ID2^hi^EOMES^lo^T-bet^int^ and ProT-NK D21 are ID2^hi^EOMES^lo^T-bet^hi^ compared to PB-NK cells, which are ID2^hi^EOMES^hi^T-bet^hi^. Altogether, these findings indicate that ProT-NK cells express key activation receptors (except CD16), cytotoxic molecules (perforin, granzyme B), and transcription factors ID2 and T-bet, confirming their NK identity. However, they remain less mature than PB-NK cells, as reflected by their lack of CD16, KIR molecules, and EOMES, and lower NKG2A/CD94 expression, highlighting how the unique phenotype of ProT-NK cells differs significantly from that of PB-NK cells.

### 3.3 High-dimensional characterization of ProT-NK cells

**Figure 3:**
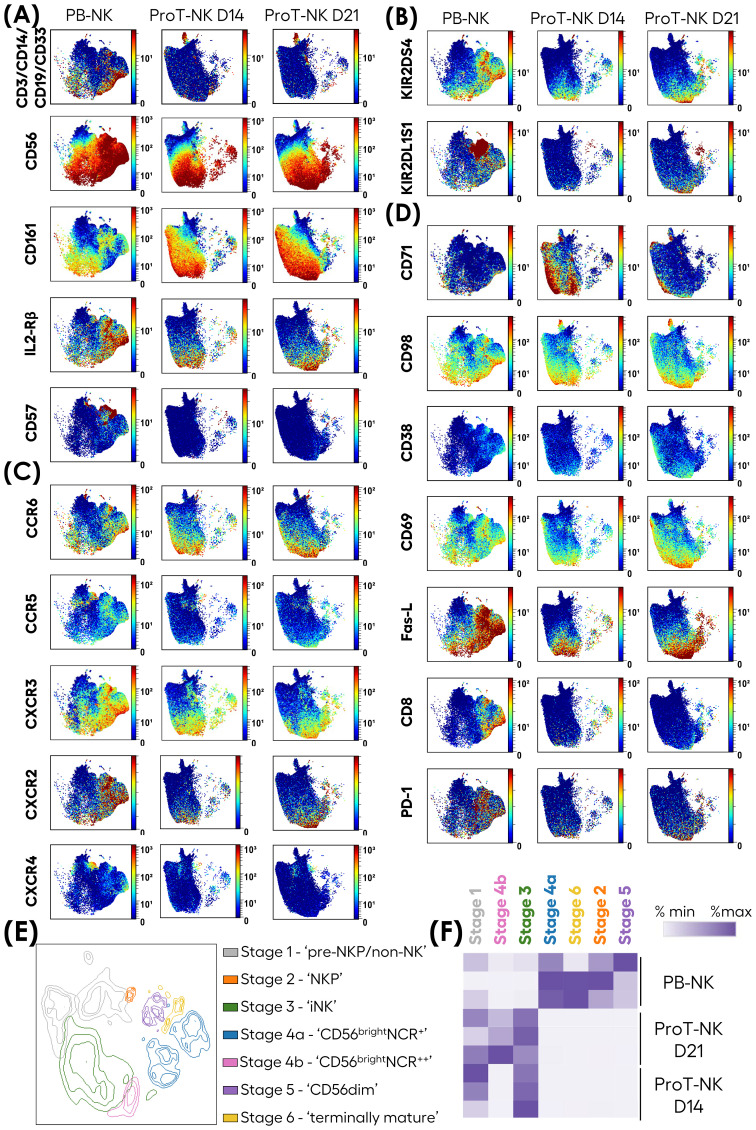
In-depth phenotypic characterization of ProT-NK cells. Mass cytometry analysis of ProT-NK D14, ProT-NK D21 and day 14 expanded PB-NK cells. **(A-D)** Expression of indicated surface proteins on viable cells and projected on UMAPs. **(A)** NK ontogeny markers. **(B)** KIRs. **(C)** Chemokine receptors. **(D)** Markers of high metabolism and activation. Merged data of different donors (N=3) for each sample are represented. **(E, F)** Merged data on viable cells from all donors (N=3) for each sample was clustered using FlowSOM. **(E)** UMAP showing seven distinct clusters, manually annotated based on CD56, CD161, CD94, NKG2D, CD57, CD16, KIRs, NCRs, and CXCR4 differential expressions. **(F)** Heatmap of cluster abundances (% of cluster) for each donor across all samples. N represents the number of donors.

Given the distinct phenotype of ProT-NK cells revealed by flow cytometry, we further explored their characteristics in-depth through cytometry by time of flight (CyTOF), using two extensive NK-related panels: one focused on phenotype and other on function. We analyzed three donors per condition (PB-NK, ProT-NK D14, and ProT-NK D21) and used UMAP (42) to visualize cell populations based on protein expressions (**Figure 3**, **Supplementary Figure S4**). Most of the ProT-NK D14 and D21 cells expressed high levels of CD56 and CD161, with no contamination of other cell lineages (as indicated by lack of expressions of CD3, CD14, CD19 and CD33) (**Figure 3A**), confirming their NK identity and purity, consistent with flow cytometry results. PB-NK cells expressed lower levels of CD161 compared to ProT-NK D14 and D21 (**Figure 3A**). IL-2Rβ and CD57 were lower in ProT-NK cells compared to PB-NK cells (**Figure 3A**). KIR2DS4, an activating KIR, was expressed in both ProT-NK D14 and D21, with ProT-NK D21 showing similar levels of expression to PB-NK cells (**Figure 3B**). KIR2DL1S1 was expressed at low levels across all conditions (**Figure 3B**). Other activation markers showed the following: NKG2D expressions in ProT-NK cells were comparable to PB-NK. CD16 expression was absent in ProT-NK, contrary to PB-NK, which exhibited a distinct subpopulation expressing CD16 (**Supplementary Figures S4A, B**). DNAM-1 was comparable with ProT-NK D21 and PB-NK but lower in ProT-NK D14 (**Supplementary Figures S4A, B**). NCRs (NKp30, NKp44, NKp46) expression patterns were similar across ProT-NK and PB-NK (**Supplementary Figures S4A, B**). KLRG1 and NKG2A/CD94 heterodimer were detected in all samples but at higher levels in PB-NK (**Supplementary Figures S4A, C**). Inhibitory KIRs (KIR2DL2L3, KIR3DL1) were low or absent in ProT-NK (**Supplementary Figures S4A, C**). Importantly, chemokine receptor expression analysis revealed high CCR6 and CXCR3 expressions in ProT-NK D14 cells, comparable to PB-NK, while the expressions of other chemokine receptors (CCR5, CXCR2, CXCR4) were higher in PB-NK. Notably, CXCR4 expression was almost absent in all conditions (**Figure 3C**). The transferrin receptor (CD71), which is known to be related to enhanced cell metabolism and growth (49, 50), was strongly expressed by ProT-NK D14, while PB-NK and ProT-NK D21 showed lower relative expression, suggesting enhanced metabolism and growth of ProT-NK D14 cells (**Figure 3D**). Moreover, comparable to PB-NK, ProT-NK D14 and D21 showed high expressions of CD98 (**Figure 3D**), which is already shown to be upregulated in metabolically active NK cells (51–53). Interestingly, CD38, reported to be associated with fratricide and exhaustion (54, 55) was not as higly expressed by ProT-NK cells than by PB-NK. CD69 and Fas-L were expressed both by ProT-NK and PB-NK cells. Fas-L was expressed at higher levels by PB-NK than by ProT-NK cells (**Figure 3D**). Finally, when CD8 expression was not detected in ProT-NK cells, PD-1 was minimally expressed in ProT-NK D21 likely due to the extended culture time (**Figure 3D**).

NK cell development progresses through defined stages that impact their function (56). To elucidate these stages within our ProT-NK cell product, we applied FlowSOM clustering on merged PB-NK, ProT-NK D14, and ProT-NK D21 data (**Figure 3E**). FlowSOM generated seven clusters (**Figure 3E**), and based on the expressions of CD56, CD161, CD94, NKG2D, CD57, CD16, KIRs, natural cytotoxicity receptors (NCRs), and CXCR4 we correlated them with already established NK cell developmental stages (63–65). Analysis of the clusters by manually annotating them revealed the presence of all NK cell developmental stages, from pre-NKP to terminally mature NK cells. Cluster abundance in PB-NK, ProT-NK D14, and ProT-NK D21 was then assessed (**Figure 3F**). ProT-NK D14 was enriched in stage 1 (pre-NKP/non-NK: CD56^lo^CD161^+^CD94^-^NKG2D^-^) and stage 3 (iNK: CD56^+^CD161^bright^CD94^+^NKG2D^+^CD16^-^), while ProT-NK D21 showed an increased presence of stage 4b cells (CD56^bright^: CD161^+^CD94^+^NKG2D^+^NCRs^++^) in addition to stage 1 and 3, suggesting an activated state after long cytokine exposure. PB-NK cells displayed higher abundance of mature clusters, including stages- 2 (NKP: CD56^+^CD161^-^CD94^+^NKG2D^+^), 4b (CD56^bright^: CD161^+^CD94^+^NKG2D^+^NCRs^+^), 5 (CD56^dim^: CD161^+^CD94^+^NKG2D^+^NCRs^++^CD16^+^KIR^++^CD57^-^) and 6 (terminally mature: CD56^dim^CD161^-^CD94^+^NKG2D^+^NCRs^++^CD16^+^KIR^++^CD57^hi^). Overall, these results show distinct maturation stages in ProT-NK cells, with early markers in ProT-NK D14 and more mature features in ProT-NK D21, highlighting their unique developmental profile compared to PB-NK cells.

### 3.4 ProT-NK hold functional and cytotoxic potential

**Figure 4:**
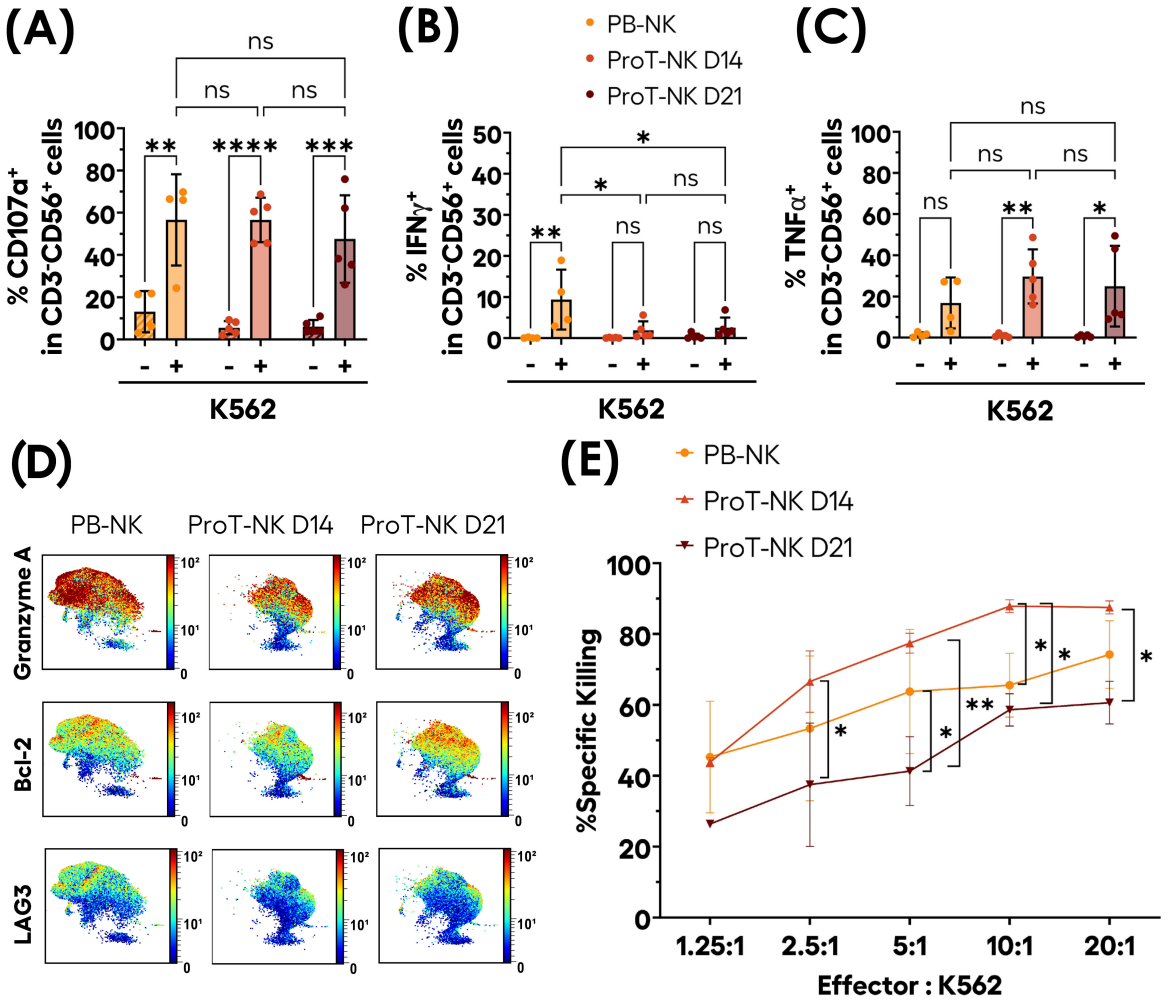
Functional activity of ex-vivo produced ProT-NK cells. **(A-C)** ProT-NK D14, ProT-NK D21 and day 14 expanded PB-NK cells were stimulated or not with K562 cells at 1:2 ratio of NK to K562 cells for 6 hours. After co-incubation, the cells were analyzed by flow cytometry for degranulation (based on CD107a surface expression) and cytokine induction (TNFα and IFNγ expression). Graphs representing mean expression frequencies of CD107a **(A)**, IFNγ **(B)**, and TNFα **(C)** within CD3^-^CD56^+^ cells (mean ± SD, PB-NK N=4, ProT-NK D14 N=5, ProT-NK D21 = 5). **(D)** UMAP plots showing the expression of indicated intracellular proteins in CD56^+^ cells as analyzed by mass cytometry for ProT-NK D14, ProT-NK D21 and day 14 expanded PB-NK cells after stimulation for 6 hours with K562 cells at 1:2 ratio of NK to K562 cells. Merged data of different donors (N=3) for each sample are represented. **(E)** Level of cytotoxicity of ProT-NK D14, ProT-NK D21 and day 14 expanded PB-NK cells against K562 cells at the indicated effector (NK) to target (K562) ratios after co-incubation for 5 hours (mean ± SD, PB-NK N=3, ProT-NK D14 N=3, ProT-NK D21 N=3). All the statistical significances were determined by two-way ANOVA test: *p ≤ 0.05; **p ≤ 0.01; ***p ≤ 0.001; ****p ≤0 .0001. ns, non-significant. N represents the number of donors.

Subsequently, we assessed the functional and cytotoxic potential of ProT-NK cells. First, we focused on their degranulation ability, a key function of NK cells that involves releasing their cytolytic granules (e.g. perforin and granzyme B) into the extracellular space (57). The NK cells were stimulated with K562 cells for 6 hours and the degranulation was detected by surface expression of CD107a. Without stimulation, PB-NK cells showed spontaneous degranulation at low percentages (13.1 ± 9.8% CD107a^+^ (mean ± SD)), and so did ProT-NK (ProT-NK D14: 5.5 ± 3.1% CD107a^+^, ProT-NK D21: 6 ± 3.2% CD107a^+^) (**Figure 4A**, **Supplementary Figure S5A**). When stimulated with K562, ProT-NK cells degranulated efficiently (ProT-NK D14: 56.5 ± 10.5% CD107a^+^, ProT-NK D21: 47.5 ± 20.7% CD107a^+^ (mean ± SD)), at levels comparable to PB-NK cells (56.6 ± 21.6%) (**Figure 4A**, **Supplementary Figure S5A**).

Next, we analyzed cytokine production in response to K562 cell stimulation and showed that ProT-NK cells produced low levels of IFNγ (ProT-NK D14: 1.9 ± 2.2%, ProT-NK D21: 2.5 ± 2.5%), significantly lower than produced by PB-NK cells (9.4 ± 7.3%) (**Figure 4B**, **Supplementary Figure S5B**). Interestingly, ProT-NK cells showed higher TNFα production compared to PB-NK cells (PB-NK: 16.9 ± 12.4%, ProT-NK D14: 29.7 ± 13.1%, ProT-NK D21: 25 ± 19.6%), although this difference was not statistically significant (**Figure 4C**, **Supplementary Figure S5C**).

Mass cytometry analysis of ProT-NK and PB-NK cells after stimulation with K562 cells demonstrated their high expressions of the cytotoxic molecules Granzyme A, Granzyme B and Perforin, with PB-NK showing higher levels of Granzyme A, Granzyme B, Perforin (**Figure 4D**, **Supplementary Figures S5D, E**). They produced both TNFα and IFNγ with much lower IFNγ levels in ProT-NK cells than in PB-NK, confirming the FC results (**Supplementary Figures S5D, E**). Bcl-2, an anti-apoptotic protein, was significantly higher in both ProT-NK D14 and D21 compared to PB-NK (**Figure 4D**, **Supplementary Figure S5D**). LAG-3, which is known to be associated with immune cell exhaustion (58), was expressed at similar levels in PB-NK and ProT-NK D21, while ProT-NK D14 exhibited lower levels (**Figure 4D**, **Supplementary Figure S5D**), indicating a less exhausted phenotype.

The cytotoxic activity of ProT-NK cells was further studied using K562 cells as target cells. After co-incubation for 5 hours at various effector-to-target ratios (**Figure 4E**, **Supplementary Figure S5E**), ProT-NK D14 and PB-NK showed similar level of target cell killing at 1.25:1 ratio (PB-NK: 45.3 ± 15.8%; ProT-NK D14: 43.8 ± 1%, mean ± SD), while ProT-NK D21 showed a comparatively low level of killing (26.4 ± 0.1%). At 2.5:1 ratio, although not significant, target cell killing by ProT-NK D14 was higher (66.6 ± 8.7%) than PB-NK (53.3 ± 20.5%) and significantly greater than ProT-NK D21 (37.5 ± 17.4%). ProT-NK D14 consistently showed significantly superior killing than PB-NK and ProT-NK D21 when effector-to-target ratios were increased from 5:1 to 20:1 reaching a plateau of killing at 10:1. These results showed that both ProT-NK D14 and D21 could kill K562 cells with ProT-NK D14 displaying greater potency than that of ProT-NK D21. Remarkably, ProT-NK D14 was as cytotoxic as or more cytotoxic than PB-NK cells. In sum, ProT-NK cells, especially at day 14, possess functional and cytotoxic potential, efficiently killing target cells *in vitro*.

### 3.5 ProT-NK cells can home, persist and mature *in vivo*

**Figure 5:**
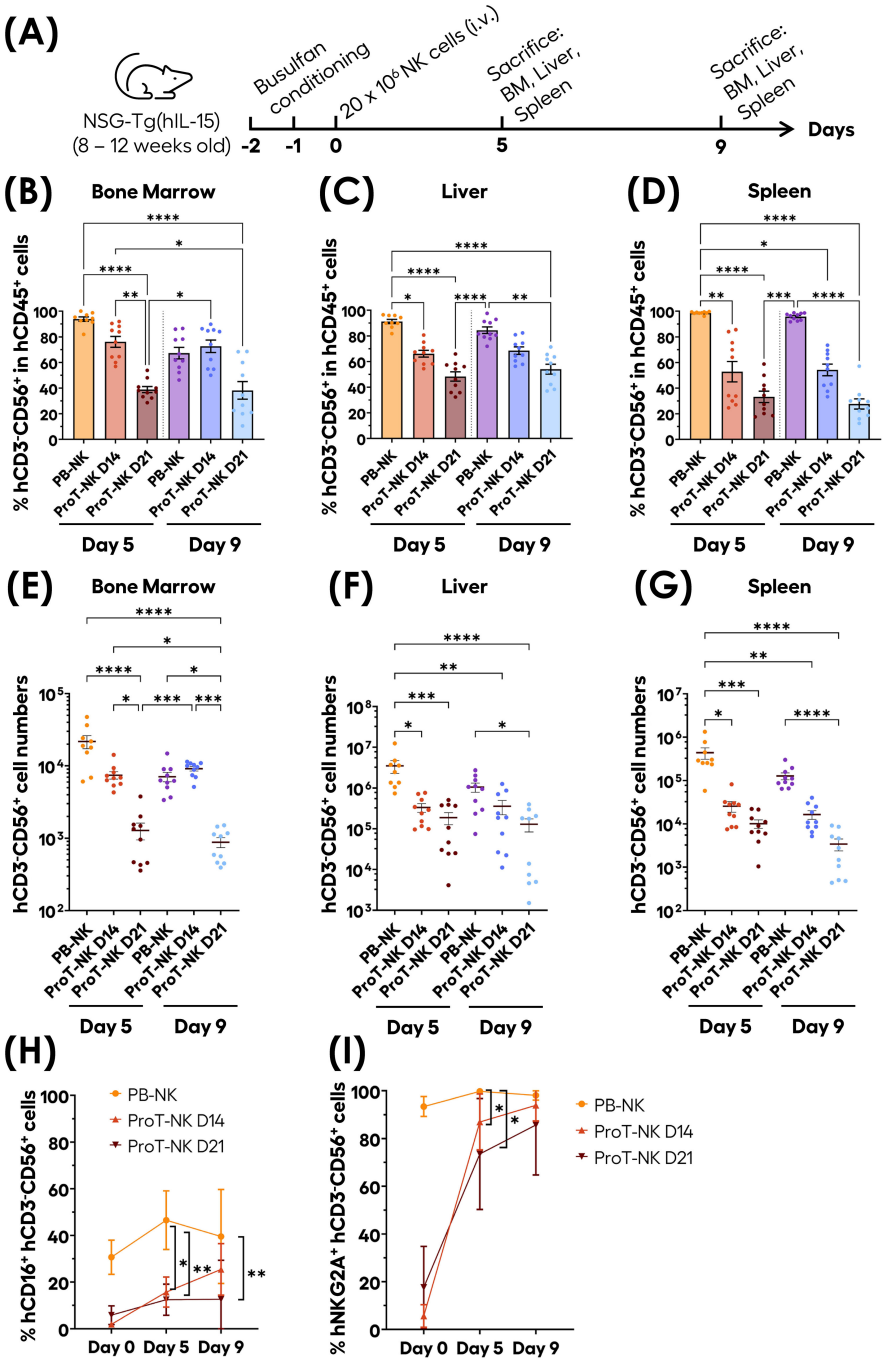
*In vivo* homing, persistence and maturation capacities of ProT-NK cells in NSG-Tg(IL-15) mouse model. **(A)** Schematic representation of the experimental procedure: Adult NSG-Tg(hIL-15) mice were conditioned on days -2 and -1 with Busulfan (total dose of 30 mg/kg) and injected intravenously with 20 x 10^6^ NK cells (PB-NK, ProT-NK D14, or ProT-NK D21) on day 0. Bone marrow (BM), liver, spleen and blood were analyzed on day 5 and 9 post-injection. **(B-D)** Graphical representation of the mean frequencies of hCD3^-^CD56^+^ within hCD45^+^ in BM **(B)**, liver **(C)** and spleen **(D)** at 5 and 9 days after injection (mean ± SD, PB-NK N=9, ProT-NK D14 N=10, ProT-NK D21 = 10). **(E-G)** Graphs showing the mean cell number of CD3^-^CD56^+^ in BM **(E)**, liver **(F)** and spleen **(G)** on day 5 and day 9 after injection (mean ± SD, PB-NK N=9, ProT-NK D14 N=10, ProT-NK D21 = 10). **(H, I)** Mean expression frequencies of hCD16 **(H)** and hNKG2A **(I)** within CD3^-^CD56^+^ cells in BM on day 5 and 9 post NK cell injection (mean ± SD, PB-NK N=9, ProT-NK D14 N=10, ProT-NK D21 = 10). The statistical significances were determined by Kruskal-Wallis test: *p ≤ 0.05; **p ≤ 0.01; ***p ≤ 0.001, ****p ≤ 0.0001. N represents the number of mice. Each dot represents a recipient mouse.

To further explore the homing potential and persistence of ProT-NK cells, we administered them in a murine model. Murine IL-15 cross-reacts poorly with human IL-15 receptors. To better replicate human NK cell biology, we used NSG-Tg(hIL-15) mice, which express human IL-15 at physiological levels and support NK cell differentiation after CD34^+^ HSCT (59, 60). This model is pertinent to evaluate both the homing ability and *in vivo* differentiation and persistence of ProT-NK cells. Adult NSG-Tg(hIL-15) mice were conditioned with busulfan (30 mg/kg). One day after conditioning, NK cells (PB-NK, ProT-NK D14 or ProT-NK D21) were injected intravenously at a single dose of 20 x 10^6^ CD3^-^CD56^+^ cells per mouse. Bone marrow, liver and spleen were analyzed at 5 and 9 days post-injection (**Figure 5A**), applying the gating strategy detailed in **Supplementary Figure S6A**.

Human CD45^+^ and CD3^-^CD56^+^ cells were detected in the bone marrow (BM), liver, and spleen in all groups (PB-NK, ProT-NK D14, ProT-NK D21), on both days, confirming that ProT-NK cells can home to both hematopoietic and non-hematopoietic organs in NSG-Tg(hIL-15) mice (**Figures 5B-D**). In the BM, hCD56^+^ cells were detected at comparable frequencies in ProT-NK D14 and PB-NK injected mice on day 5 (93.8 ± 5.3% for PB-NK and 76.1 ± 13.5% for ProT-NK D14 (mean ± SD)) (**Figure 5B**, **Supplementary Figures S6B, C**). By day 9, PB-NK levels decreased (67.3 ± 14.2%), whereas ProT-NK D14 levels remained stable (72.6 ± 15.5%) suggesting that ProT-NK D14 cells persist in the BM up to 9 days after intravenous injection. ProT-NK D21 levels in BM were significantly lower (38.8 ± 7.5%) than those of ProT-NK D14 on day 5 (76.1 ± 13.5%) and remained stable through day 9 (38.2 ± 21.7%) (**Figure 5B**). A similar pattern was observed in liver (**Figure 5C**) and spleen (**Figure 5D**), where on day 5, PB-NK cells showed significantly higher hCD56^+^ frequencies (91.2 ± 4.9% liver, 98.6 ± 1.1% spleen) compared to ProT-NK D14 cells (66.1 ± 8.3% liver, 52.7 ± 25.1% spleen). By day 9, ProT-NK D14 levels remained lower than those of PB-NK but the difference was no longer significant (**Figures 5C, D**). ProT-NK D21 cells showed consistently lower frequencies on day 5 in both the liver (48.3 ± 11.6%) and spleen (33.2 ± 14.2%) compared to both PB-NK and ProT-NK D14 cells, and these levels remained stable by day 9 (**Figures 5C, D**). When analyzing hCD3^-^CD56^+^ cell numbers in BM (**Figure 5E**) we observed that NK cell mean number was 3-fold higher in PB-NK cell injected mice on day 5 post transplantation (2.2 ± 0.4 x 10^4^ cells, mean ± SEM) compared to mice injected with ProT-NK D14 (7428 ± 877). By day 9, the numbers were similar between PB-NK and ProT-NK due to a decrease in PB-NK, (7067 ± 1051) and an increase in ProT-NK D14 numbers (9144 ± 641). This suggests greater persistence and expansion capacity for ProT-NK D14 cells than for PB-NK cells in the BM. NK cell numbers in ProT-NK D21 condition slightly decreased from day 5 (1280 ± 332) to day 9 (877 ± 137), indicating their limited persistence (**Figure 5E**). A similar trend was observed in cell numbers for both liver (**Figure 5F**) and spleen (**Figure 5G**). On day 5, PB-NK cell injected mice exhibited a significantly higher numbers–by 10-fold–of hCD3^-^CD56^+^ cells (3.5 ± 1.2 x 10^6^ cells in the liver and 4.4 ± 1.3 x 10^5^ cells in the spleen) compared to ProT-NK D14 group (3.3 ± 0.8 x 10^5^ cells in the liver and 2.6 ± 0.6 x 10^4^ cells in the spleen). By day 9, this difference was no longer statistically significant in either organ, though NK cell numbers in ProT-NK D14 group remained lower than those of PB-NK group (**Figures 5F, G**). ProT-NK D21 cell injected mice consistently showed lower hCD3^-^CD56^+^ numbers on day 5 in both liver (1.9 ± 0.6 x 10^5^ cells) and spleen (1 ± 0.2 x 10^4^ cells) compared to PB-NK and ProT-NK D14 conditions, with the numbers remaining stable or lowering through day 9 (**Figures 5F, G**).

We evaluated the maturation of ProT-NK cells *in vivo* by assessing the kinetics of CD16 and NKG2A expression in BM (**Figures 5H, I**). CD16 is expressed mainly by CD56^dim^ mature NK cells (61, 62), while NKG2A/CD94 heterodimer is expressed on CD56^bright^ immature NK cells (56). Interestingly, while hCD16 expression of NK cells in BM remained stable for PB-NK cell injected mice from day 5 (mean ± SD, 46.5 ± 12.5%) to day 9 (39.6 ± 20.1%), its expression was increased significantly for ProT-NK D14 group (15.7 ± 6.4% for day 5 and 25.4 ± 11.1% for day 9). However, CD16 expression showed no significant change for ProT-NK D21 group from day 5 (12.4 ± 6.7%) to day 9 (12.6 ± 16.7%) (**Figure 5H**). NKG2A expression in NK cells spiked significantly for ProT-NK D14 and D21 conditions from day 0 (5.6 ± 4.7%, 17.6 ± 17.2% respectively) to day 5 (87 ± 11.7%, 73.5 ± 23.2% respectively) and remained high until day 9 (93.9 ± 6.5%, 85.7 ± 21% respectively), reaching the expression level of PB-NK cells, which had already high levels ex vivo (**Figure 5I**). This data indicates that ProT-NK cells, which were immature before injection, can mature *in vivo* in the BM of NSG-Tg(hIL-15) mice. These findings demonstrate that the homing, persistence, and maturation capacities of ProT-NK cell, particularly D14, enable it to outperform the ProT-NK D21 product. ProT-NK D14 cells were thus chosen for further investigation of their functional potential *in vivo*.

### 3.6 *In vivo* anti-tumor activity of ProT-NK cells

**Figure 6:**
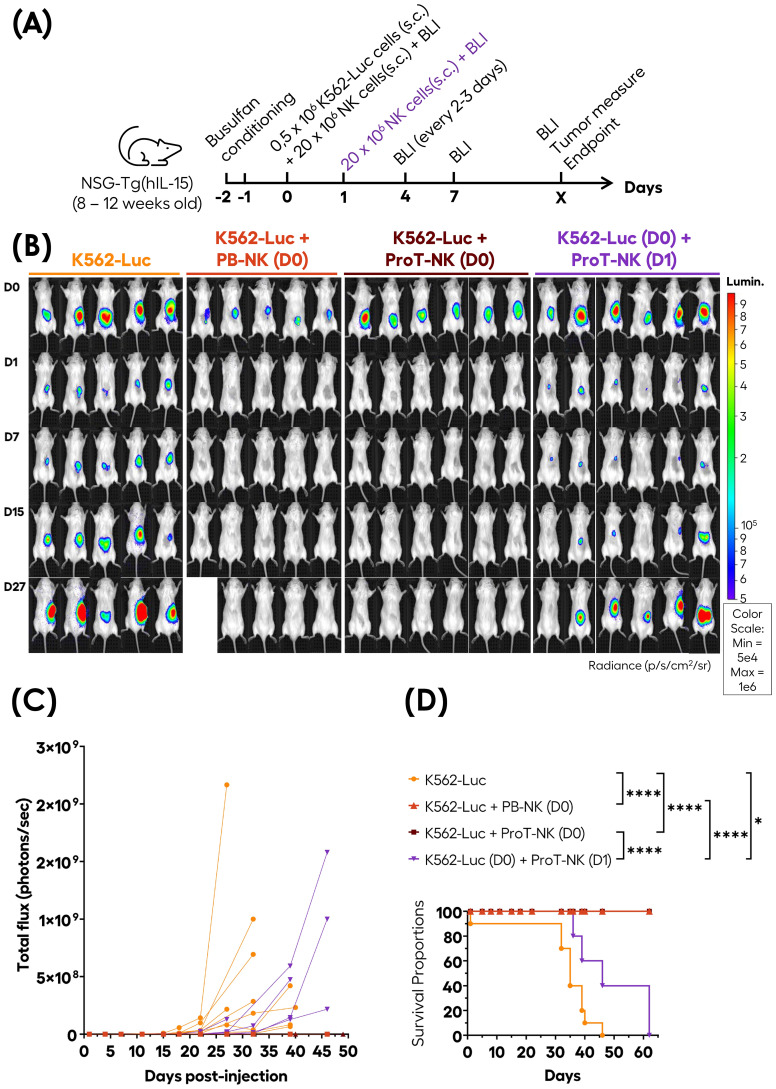
*In vivo* anti-tumor activity of ProT-NK cells in the NSG-Tg(hIL-15) mouse model. **(A)** Schematic representation of the experimental procedure: adult NSG-Tg(hIL-15) mice were conditioned on days -2 and -1 with Busulfan (total dose of 30 mg/kg) and subcutaneously (s.c.) injected on day 0 (D0) with K562-Luc alone (0.5 x 10^6^ cells/mouse), K562-Luc mixed with control PB-NK (20 x 10^6^ cells/mouse) or ProT-NK D14 (20 x 10^6^ cells/mouse). One group of mice injected with K562-Luc alone on day 0 received ProT-NK D14 (20 x 10^6^ cells/mouse) on day 1 (D1) subcutaneously around the site of tumor cell injection, indicated by violet color. Tumor growth was measured based on the intensity of bioluminescence detected with bioluminescence imaging (BLI). **(B)** BLI images captured at the indicated time points. Blue to red colors represent the intensity of bioluminescence (red, highest; blue, lowest). Lumin.: Luminescence. **(C)** Tumor load with time plotted as individual repeated measures, with each replicate connected. Two independent experiments were performed for K562 alone and K562 (D0) + ProT-NK (D0) group, with similar outcomes. K562-Luc: N=9 (orange lines); K562 + PB-NK (D0) (red lines): N=5; K562 + ProT-NK (D0) (brown lines): N= 11; K562 (D0) + ProT-NK (D1) (violet lines): N= 6. **(D)** Kaplan Meier plots showing the probability of survival. P values were computed using the log-rank (Mantel-Cox) test: *p ≤ 0.05; ****p ≤ 0.0001. One mouse from the K562 + PB-NK (D0) group died at day 15 after luciferin injection and was excluded from the survival analysis. N represents the number of mice.

We established a human tumor xenograft model in NSG-Tg(hIL-15) mice by subcutaneous injection of K562 luciferase expressing cells (K562-Luc) to study the *in vivo* anti-tumor activity of ProT-NK D14 cells. NSG-Tg(hIL-15) mice (8 -12 weeks old) were conditioned with low dose busulfan for two consecutive days before NK injection. On day 0, mice received a subcutaneous (s.c.) injection of K562-Luc alone (0.5 x 10^6^ cells/mouse), or a mix (before injection) of K562-Luc and PB-NK or ProT-NK D14 (20 x 10^6^ cells/mouse). The tumor and NK cells were mixed immediately before injection under cold conditions, ensuring minimal contact time (3 minutes) to prevent pre-injection cytotoxicity. Another group received ProT-NK D14 (20 x 10^6^ cells/mouse) one day after K562-Luc injection (0.5 x 10^6^ cells/mouse), around the tumor site (**Figure 6A**). Tumor growth was monitored over time using bioluminescence imaging (BLI) with the IVIS^®^ Spectrum system.

Remarkably, while tumor growth, indicated by increased bioluminescence, was observed in mice injected with K562-Luc alone, no tumor growth (or bioluminescence increase) was detected in mice co-injected with K562-Luc and either PB-NK cells or ProT-NK cells at all analyzed time points (up to 62 days) (**Figures 6B, C**). Both PB-NK and ProT-NK cells eliminated K562-Luc cells subcutaneously within 24 hours, as shown by bioluminescence acquisition at day 1. Interestingly, delayed ProT-NK injection slowed down tumor (**Figures 6B, C**, violet lines), suggesting that ProT-NK cells can kill tumor cells *in vivo*. Additionally, all mice injected with K562-Luc cells alone reached the endpoint (tumor size ≥ 1.7 cm in diameter) and were sacrificed between day 32 and day 46. In contrast, 100% of the mice co-injected with PB- or ProT-NK cells survived until the last analysis point (day 62) (**Figure 6D**). Notably, delayed ProT-NK injection significantly extended survival compared to the untreated group (p=0.0295) and were sacrificed between day 35 and day 60. This data implies that ProT-NK cells possess antitumor potential *in vivo*.

### 3.7 Generation of CAR-ProT-NK cells with proprietary ex vivo feeder-free culture system

**Figure 7:**
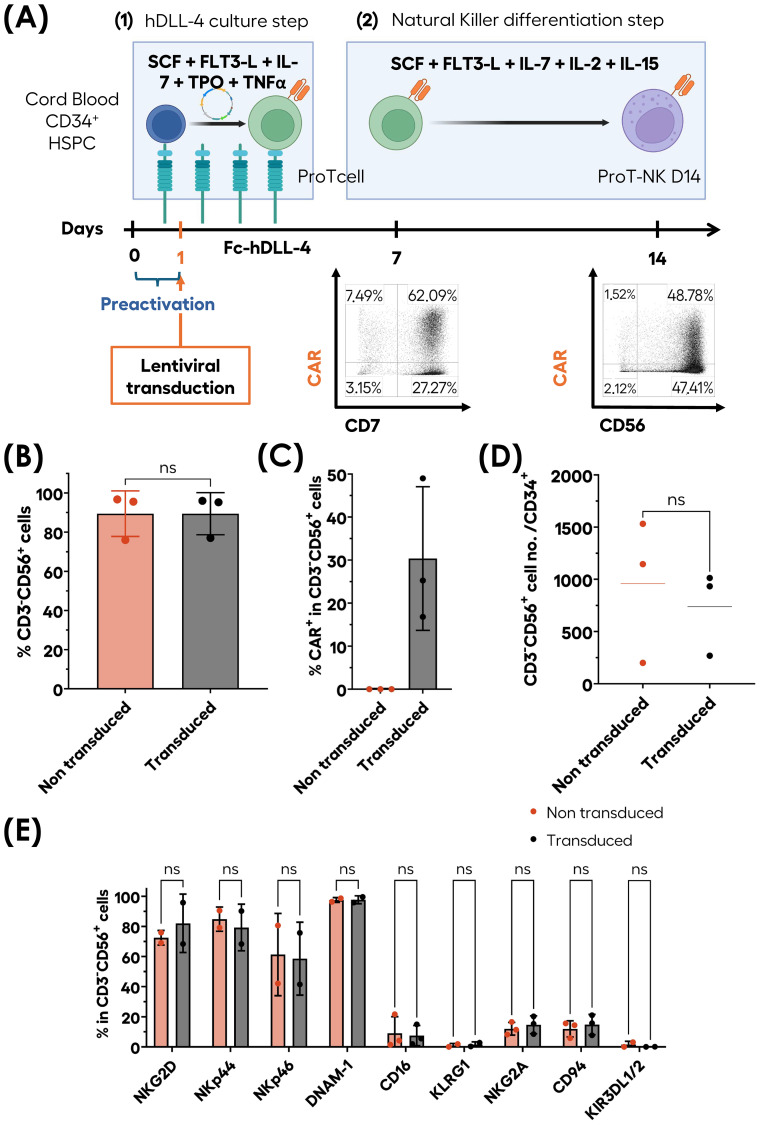
Ex-vivo generation of CAR-ProT-NK cells. CB CD34^+^ cells (blue cells) were pre-activated for 12 hours and subsequently transduced for 6 hours under hDLL4 culture conditions. Following transduction, the cells were further cultured under hDLL4 culture until day 7 to produce transduced ProTcells (green cells). These transduced ProTcells were then differentiated into transduced NK cells (violet cells) in feeder cell-free culture system for an additional 7 days. After a total of 14 days, the cell cultures were analyzed for phenotype and cell numbers using flow cytometry (FC). **(A)** Schematic representation of transduced ProT-NK (violet cells with orange CAR) differentiation and expansion process and representative FC plots showing CAR^+^CD56^+^ generated populations. Graphs showing the mean frequencies of CD3^-^CD56^+^ cells **(B)**, CAR expression frequency within CD3^-^CD56^+^ cells **(C)** and cell number per CD34^+^ cells **(D)** after 14 days compared with non-transduced (NT) ProT-NK D14 cells from the same donor (mean ± SD, N=3). **(E)** Flow cytometry characterization of activation and inhibitory NK membrane markers expressed by transduced ProT-NK generated products compared with NT ProT-NK (mean ± SD, N=2). Statistical significance was assessed using two-tailed unpaired t-test: ns: non-significant. N represents the number of donors.

To determine whether genetically modified transduced ProT-NK could be generated in our ex-vivo feeder-cell-free culture system, CB HSPCs were pre-activated on immobilized hDLL4 culture supplemented with a cytokine cocktail (**Figure 7A**), transduced with a lentiviral vector encoding an anti-CD19 chimeric antigen receptor (CAR) (FMC63), and cultured for 7 days in the hDLL4 culture phase. After 7 days, the resulting ProTcells (>85% CD7^+^) were cultured in a second feeder cell-free NK differentiation and expansion phase with cytokines and were analyzed on day 14 (**Figure 7A**). The ProTcells from CAR-transduced condition efficiently differentiated into ProT-NK cells (CD3^-^CD56^+^), achieving purity levels over 80% in the final product (**Figures 7A, B**) with an average of 30.4 ± 16.7% of CAR expressing ProT-NK cells (**Figure 7C**). No significant differences were observed in the NK cell frequencies between non-transduced (NT) (mean ± SD, 89.4 ± 11.6% of CD3^-^CD56^+^), and CAR-transduced condition (mean 89.4 ± 10.8% of CD3^-^CD56^+^) (**Figure 7B**). Additionally, the NK cell yields (**Figure 7D**) for NT (mean ± SEM, 959 ± 395) and CAR-transduced conditions (738 ± 236) showed no significant difference, indicating that the transduction step does not impair NK differentiation and expansion in this culture system. Surface marker characterization (**Figure 7E**) revealed that ProT-NK cells derived from CAR-transduced condition expressed similar levels of activation receptors (NKG2D, NKp44, NKp46, DNAM-1 and CD16) as NT ProT-NK cells. Likewise, both cell types expressed comparable levels of inhibitory receptors (KLRG1, CD94/NKG2A and KIR3DL1/2). Collectively, these data demonstrate that our ex vivo culture process can generate CAR ProT-NK cells.

## 4 Discussion

Here, we found out that ProTcells can differentiate into NK cells and report an effective and scalable generation of pure CB HSPC-derived NK (ProT-NK) cells during a short period of either 14 (ProT-NK D14) or 21 (ProT-NK D21) days using a novel and optimized 2-step feeder cell-free culture system: a 1^st^ step producing ProTcell from CB CD34^+^ HSPCs within 7 days and a 2^nd^ step producing ProT-NK cells from ProTcells during 7 or 14 days. Characterization of ProT-NK cells revealed a molecular and functional profile distinct from that of PB-NK cells, with high activation receptor levels (NKG2D, NCRs) and low inhibitory receptor expression. ProT-NK D14 cells demonstrated cytotoxic molecule expression (perforin, granzyme), degranulation, cytokine production in response to K562 cells, and superior cytotoxicity against K562 cell targets. Additionally, they were found to be superior in mouse studies in their homing, persistence, and capacity to mature. Importantly, we showed that ProT-NK D14 holds anti-tumor potential *in vivo*. Finally, we demonstrated the compatibility of our culture system with lentiviral transduction, setting the stage for future therapeutic applications.

The mechanisms driving ProTcell, which exhibit characteristics of T-cell commitment, to differentiate into NK cells remain unclear, warranting further investigation. Flow and mass cytometry revealed distinct developmental stages within ProT-NK cell cultures, with meta-clustering based on described ontogeny-related markers (63–65). The analysis revealed that ProT-NK cells are a heterogeneous mixture of NK progenitor (NKP) and immature NK cells (iNK), showing in particular a transition from iNK to CD56^bright^ stages by day 14 marked by low NCR expression and the absence of CD16 and KIRs. By day 21, ProT-NK cells exhibited a CD56^bright^ cluster with higher NCR expression, suggesting that while some activation markers can upregulate, acquiring CD16 and KIRs may require extended culture conditions. Although CD16 deficiency limits ADCC, ProT-NK cells showed functional capabilities, expressing perforin, granzyme, and TNFα, and also demonstrating cytotoxicity against K562 cells. ProT-NK cells express high levels of activating receptors like NKG2D and NKp30, while showing low levels of inhibitory receptors such as NKG2A. Functional silencing of NKG2A has shown promise in enhancing NK cell efficacy for cancer immunotherapy, though it may trigger activation-induced cell death (68, 69). Our ProT-NK cells offer a balanced alternative, expressing low levels of NKG2A and supporting both cytotoxicity and expansion. ProT-NK cells show similar characteristics to thymic NK cells, known for dual cytotoxic and cytokine-secreting roles (66–70), suggesting that they may represent a unique NK cell subtype with T cell progenitor origin.

ProT-NK cells could be of great interest in adoptive cell therapy due to their rapid production and high fold expansion rates, both of which address major challenges in NK cell therapies (22, 71). ProT-NK cells can be generated in 7 days from cryopreserved ProTcells or up to 14 days from fresh CB CD34^+^ cells, achieving scalable yields and consistent phenotype. Compared with current NK cell production methods from stem cells, which, depending on the source (38–40, 72–74), span from 28 to 100 days, ProT-NK cells offer a highly efficient alternative. Our protocol ensures low contamination levels (CD3^+^ <1%, CD14^-^CD19^-^CD33^-^) while maintaining safety standards and minimizing GvHD risk (75, 76). Lentiviral transduction achieved 30% CAR expression in ProT-NK cells without affecting their phenotype, providing a potential foundation for a CAR-NK cell therapy approach for cancer albeit the transduction efficacy needs to be improved. Thus, ProT-NK cells may represent a promising therapeutic option, especially as a cost-effective, off-the-shelf cell product (77).

Our analysis correlated higher initial CD7^+^CD161^+^ expression at the ProTcell stage with greater NK purity, highlighting donor variability in ProT-NK product. Of note, this donor variability may also account for certain discrepancies observed between flow cytometry and mass cytometry analyses for the expression of specific markers, such as DNAM-1 and NKp44. Donor variability is a critical factor in NK therapies and significantly affects treatment response (78). For instance, Rezvani’s team highlight the importance of selecting high-purity donor cells with specific criteria for optimal cord blood units (Opt-C), which exclude samples with high nucleated red blood cells or prolonged collection-to-cryopreservation times (78). They defined Opt-C by CyTOF analysis, and identified CD161-enriched clusters as favorable donors, which aligns with our findings. Thus, CD161 expression in ProTcells may serve as a valuable biomarker for predicting the purity of ProT-NK cell cultures over time, as clinical trials using NK cells generally impose a purity threshold of over 90% of CD3^-^CD56^+^ NK cells (79–81). Optimizing donor selection using new criteria may improve ProT-NK consistency and effectiveness in future studies.

ProT-NK cells exhibit specific homing patterns, primarily targeting the bone marrow, liver and spleen; those patterns are guided by chemokine receptors such as CXCR3 and CCR6 (82, 83). Expression of these receptors supports potential migration to inflamed tissues, guided by chemokines like CCL20 and CXCL9-11 (30, 84–87). Despite lacking CXCR4, ProT-NK cells may migrate to the BM thanks to hIL-15 production by BM stromal cells (59). ProT-NK’s migration to the liver and spleen aligns with studies showing NK cells’ affinity for these organs post-transfer (30, 88, 89), and correlates with their expression of CD69, typically associated with tissue-resident NK cells (90).

The superior homing capability of ProT-NK D14 cells in the bone marrow, compared to ProT-NK D21 cells, may be explained by their enhanced intrinsic homing capacity, greater survival and persistence, or a combination of both. An additional factor could be the higher immaturity of the D14 product, which may confer greater plasticity and a higher propensity to home to the bone marrow. Immature cells are often more responsive to homing signals, which might enhance their localization to this niche (91, 92). Furthermore, CyTOF analysis revealed increased expression of exhaustion markers, such as LAG-3, in the ProT-NK D21 product, suggesting that these cells may be in a more advanced exhaustion state, which could contribute to their reduced homing potential relative to ProT-NK D14 cells. This exhaustion is likely linked to the prolonged exposure of ProT-NK D21 cells to IL-15, a cytokine that has been shown to induce exhaustion in human NK cells through metabolic defects (93). These findings underscore the impact of cytokine exposure duration on the functional capacity of ProT-NK cells, emphasizing the importance of carefully optimizing culture conditions to preserve their efficacy.ProT-NK cells persist *in vivo* for up to 9 days. Although persistence in engineered therapies can last months (78, 80, 94–96), unmodified NK cells typically last around seven days, often requiring high doses or repeated injections (22, 97). For our experiments, we selected day 5 and day 9 timepoints to assess ProT-NK cell persistence within this critical window in NSG-Tg(hIL-15) mice, yet long-term monitoring remains to be tested. Repeated injections or engineered cytokine support may extend their longevity as shown for NK cells derived from other sources (22, 98).

*In vivo*, ProT-NK D14 cells show maturation in the bone marrow, marked by increased NKG2A and CD16 expression post-injection. The sustained presence of IL-15 in the NSG-Tg(hIL-15) model likely drives NKG2A upregulation (99, 100), also maintained in PB-NK cells after infusion, even though it usually decreases with terminal maturation (101). The hypothesis that ProT-NK cells initially expressing NKG2A are selectively favored for survival and subsequent maturation in the bone marrow warrants consideration. Future studies should investigate whether this level of expression is sufficient to trigger ADCC and thus combine ProT-NK cells and specific engagers (102, 103). Other strategies to enhance CD16 expression, such as the high-affinity non-cleavable variant of CD16 (hnCD16) and CRISPR/Cas9 knockouts of ADAM17, have shown promise in boosting NK cell efficacy against tumors (102, 104, 105).

ProT-NK cells exhibit potent *in vitro* cytotoxicity against K562 cells, yet this model does not fully reflect their efficacy in complex tumor environments, as K562 cells lack MHC-I and express stress ligands (106, 107). This limitation could also explain the superior cytotoxicity observed in ProT-NK D14 cells compared to D21, as earlier-stage cells may exhibit enhanced effector functions under simplified *in vitro* conditions. Additionally, their shorter exposure to pro-inflammatory cytokines during culture might contribute to their higher functionality, as prolonged cytokine exposure can disrupt metabolic homeostasis and induce an exhausted phenotype (93). Further studies using advanced 3D *in vitro* models, which better mimic the human tumor microenvironment (TME), could provide valuable insights into ProT-NK cell functionality while reducing reliance on animal studies (108–110). *In vivo*, ProT-NK D14 cells successfully halted tumor growth when co-injected with K562-Luc cells, underscoring their tumor-killing capability. However, in the delayed treatment group in which ProT-NK cells were injected one day post-tumor establishment, tumor growth resumed although delayed their growth, suggesting that effective cytotoxicity relies on direct target engagement. To tackle this, repeated infusions, shown to be beneficial in studies with PB-NK cells (96, 111), may enhance their therapeutic durability. For future studies, it will be interesting to investigate ProT-NK cell anti-tumor potential in different tumor models to further explore their therapeutic potential. In conclusion, our results highlight the importance of refining both *in vivo* models and cell delivery methods to enhance ProT-NK efficacy for potential clinical applications.

Our findings underscore the promise of ProT-NK cells as a scalable and effective option in cell-based cancer therapy, addressing key challenges in production, functionality, and therapeutic potential.

## Data Availability

The original contributions presented in the study are included in the article/**Supplementary Material**. Further inquiries can be directed to the corresponding author.
