# Supplementary material for: Feeder-cell-free system for ex vivo production of natural killer cells from cord blood hematopoietic stem and progenitor cells
Source: Front Immunol. 2025 Feb 20;16:1531736. doi: 10.3389/fimmu.2025.1531736 (PMC11883473; doi:10.3389/fimmu.2025.1531736)
Supplement: Supplementary file 1 [file DataSheet1.pdf]

Supplementary Material

1 Supplementary Figures and Tables

1.1 Supplementary Figures

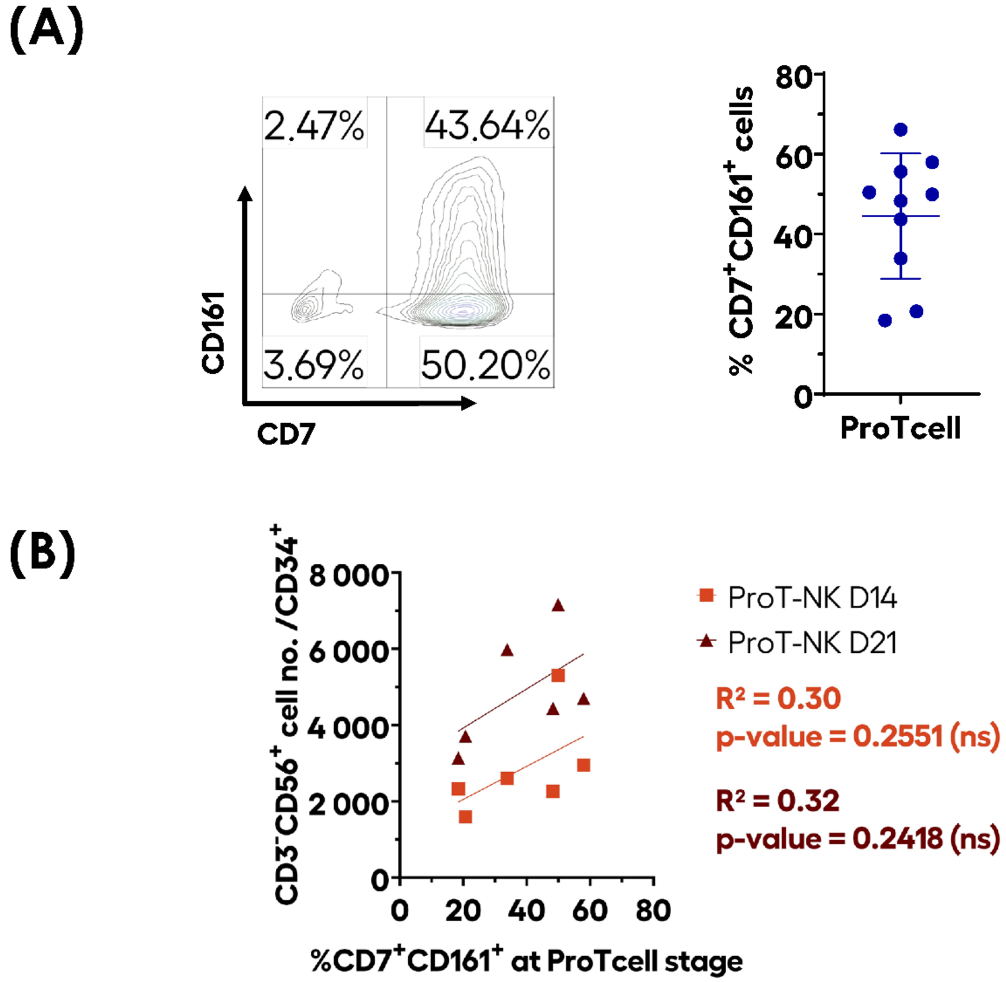

Supplementary Figure 1 – ProTcells express CD161$^+$ marker. CD34$^+$ cells isolated from CB were differentiated and expanded into ProTcells using an Fc-hDLL-4 immobilized ligand in presence of a cytokine cocktail for seven days. **(A)** Representative flow cytometry (FC) plot (left panel) of CD7$^+$CD161$^+$ expression in ProTcells, Graphs (right panel) showing the mean frequencies of CD7$^+$CD161$^+$ expression in ProTcells (mean ± SD, ProTcell N=10)**. (B)** Linear regression showing the correlation between CD161 expression at the ProTcell stage and CD3$^-$CD56$^+$ cell frequency post-NK differentiation (ProT-NK D14 N=7, ProT-NK D21 N=6). The statistical significances were determined by the Wald test for linear regression: ns: non-significant. N represents the number of donors.

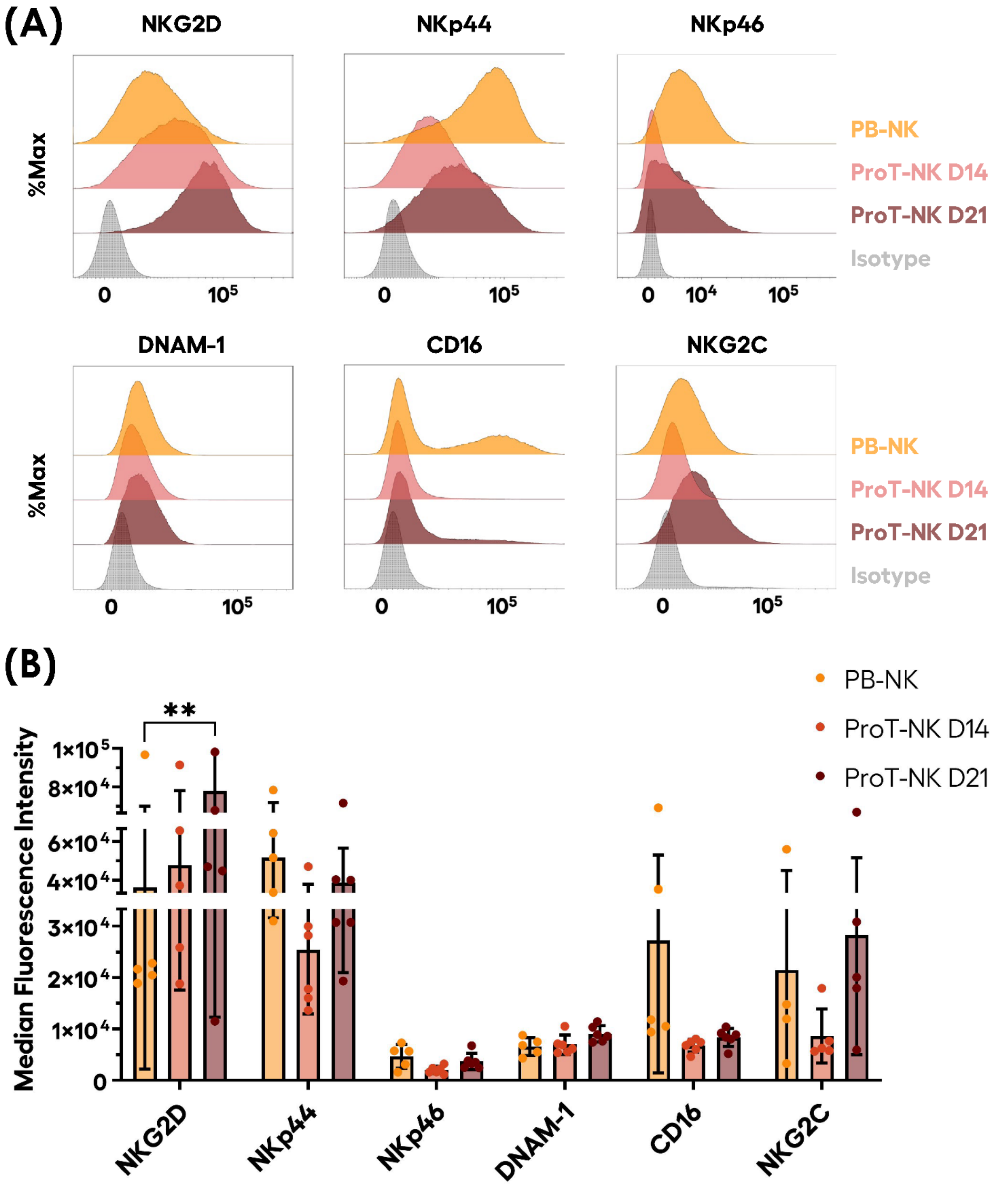

Supplementary Figure 2 – Phenotypic profile of ex vivo-generated ProT-NK cells. **(A)** Representative flow cytometry histogram showing the median fluorescence intensity (MFI) of NK activation markers. **(B)** Graphs showing MFI of activation receptor expression on $CD3^{-}CD56^{+}$ cells in ProT-NK D14, ProT-NK D21, and day 14 expanded PB-NK cells (mean± SD, N=5). The statistical significances were calculated by one-way ANOVA: $^{**}p \leq 0.01$.N represents the number of donors.

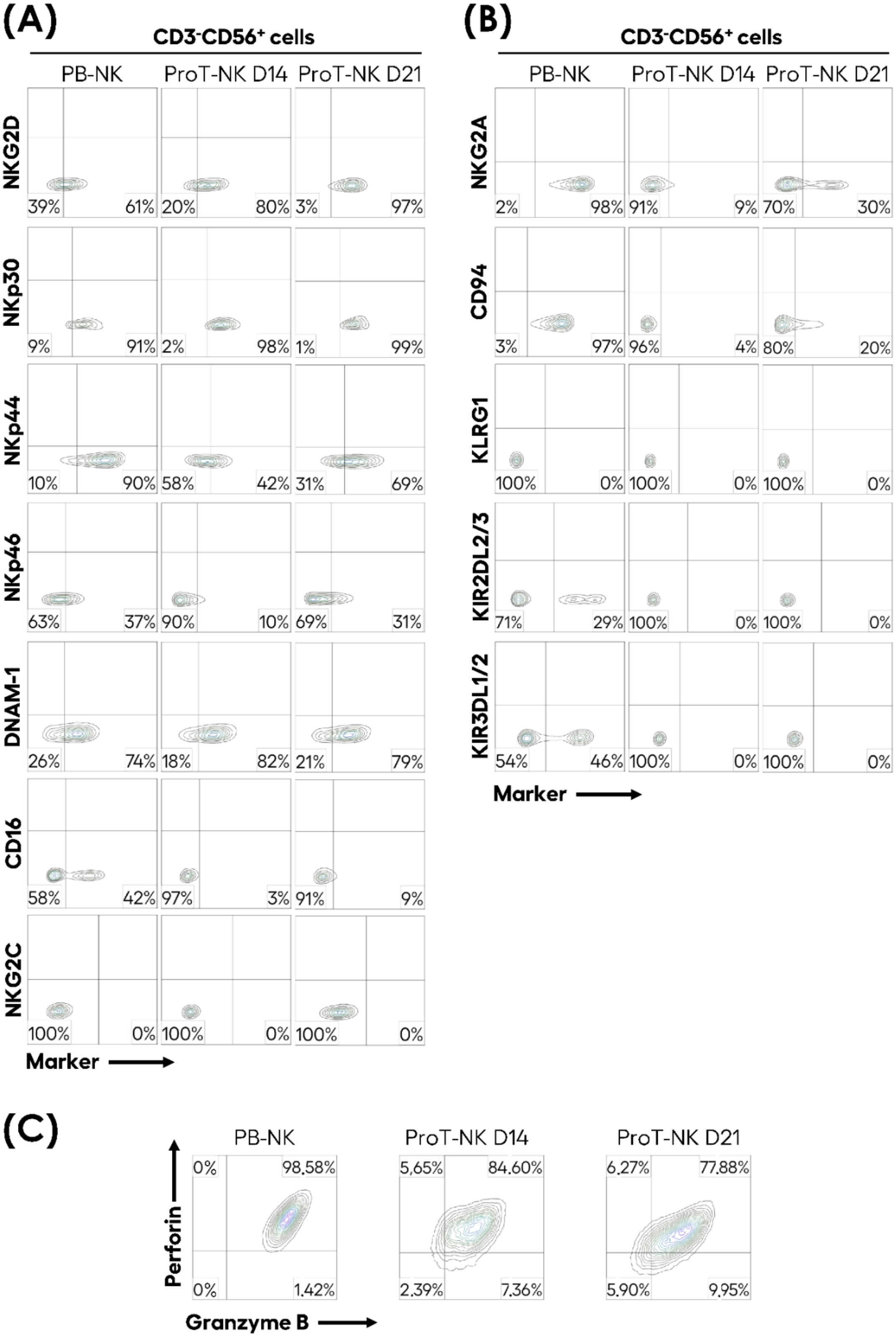

Supplementary Figure 3 – Phenotypical profile of ex-vivo generated ProT-NK cells. Representative FC plots of activation **(A)** and inhibitory **(B)** NK membrane markers expressed by $CD3^{-}CD56^{+}$ cells in ProT-NK D14 and ProT-NK D21 compared with expanded PB-NK cells. **(C)** Representative flow cytometry plots of Perforin and granzyme B co-expression among $CD3^{-}CD56^{+}$ cells in ProT-NK D14, ProT-NK D21 and day 14 expanded PB-NK cells.

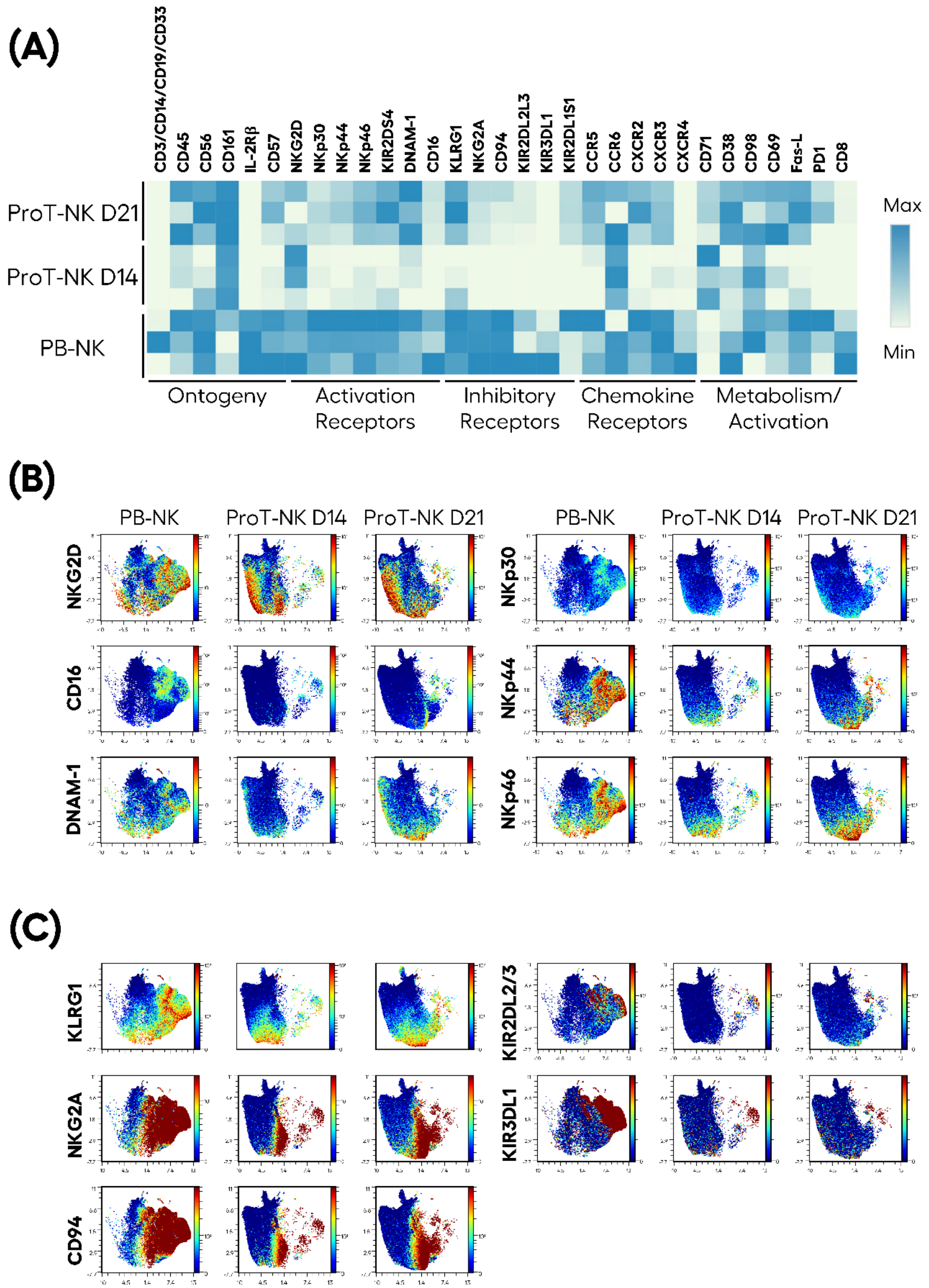

Supplementary Figure 4 – Phenotypic characterization of ProT-NK cells. (A) Heatmap displaying the median membrane protein expressions of the indicated markers on viable cells for each of the donors of all samples after mass cytometry analysis of ProT-NK D14, ProT-NK D21 and expanded PB-NK. **(B-C)** UMAPs showing the expressions of the indicated activation receptors **(B)** and inhibitory receptors **(C)**. Merged data of different donors (N=3) for each sample are represented.

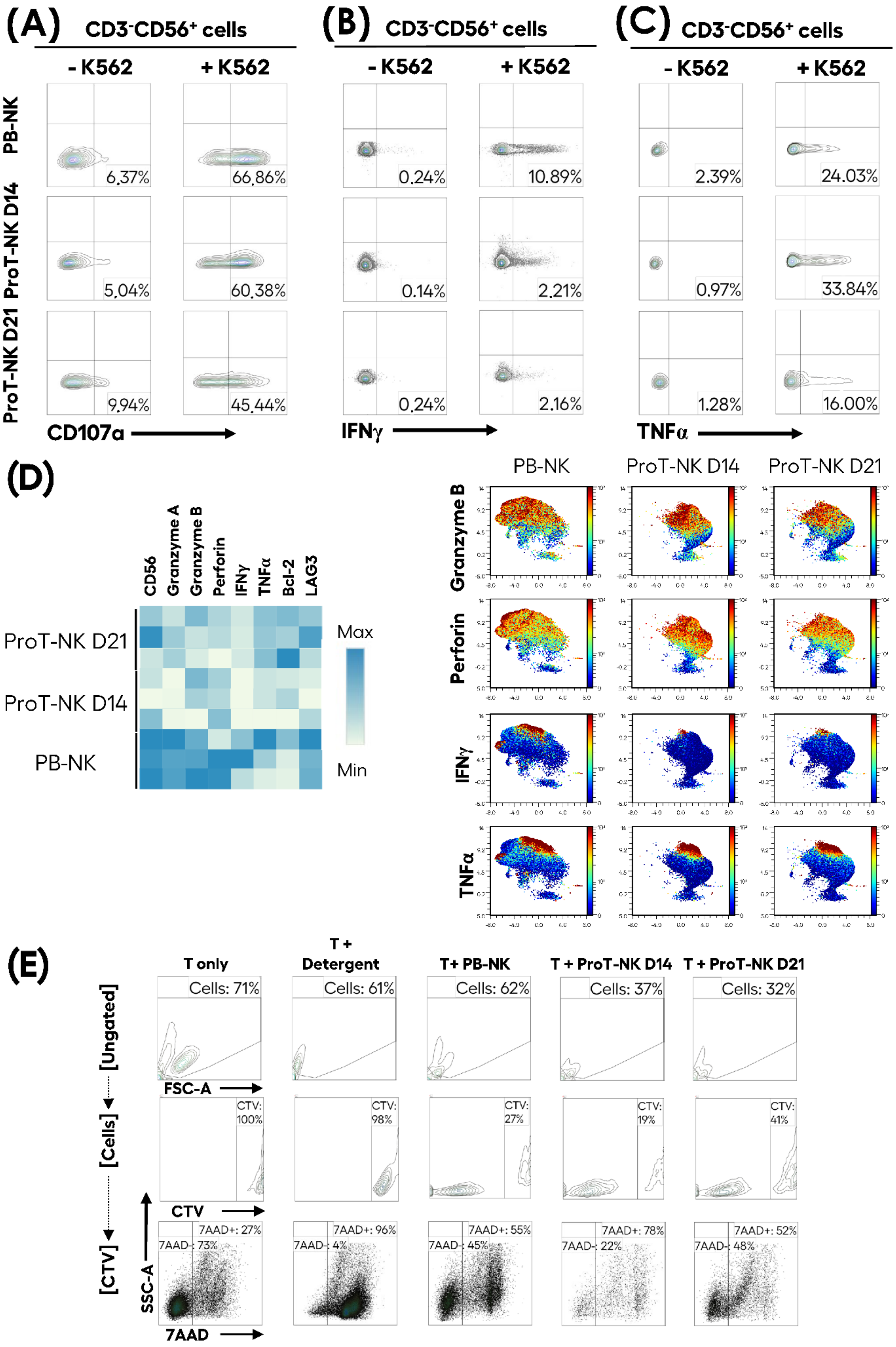
(A) CD3-CD56+ cells
- K562
+ K562
PB-NK
ProT-NK D14
ProT-NK D21
6.37%
66.86%
5.04%
60.38%
9.94%
45.44%
CD107a
(B) CD3-CD56+ cells
0.24%
10.89%
0.14%
2.21%
0.24%
2.16%
IFNγ
(C) CD3-CD56+ cells
2.39%
24.03%
0.97%
33.84%
1.28%
16.00%
TNFα
(D)
CD56
Granzyme A
Granzyme B
Perforin
IFNγ
TNFα
Bcl-2
LAG3
Max
Min
PB-NK
ProT-NK D14
ProT-NK D21
Granzyme B
Perforin
IFNγ
TNFα
(E)
T only
T + Detergent
T+ PB-NK
T + ProT-NK D14
T + ProT-NK D21
Cells: 71%
Cells: 61%
Cells: 62%
Cells: 37%
Cells: 32%
FSC-A
CTV: 100%
CTV: 98%
CTV: 27%
CTV: 19%
CTV: 41%
CTV
7AAD+: 27%
7AAD-: 73%
7AAD+: 96%
7AAD-: 4%
7AAD+: 55%
7AAD-: 45%
7AAD+: 78%
7AAD-: 22%
7AAD+: 52%
7AAD-: 48%
SSC-A
7AAD
[Ungated]
[Cells]
[CTV]

Supplementary Figure 5 - Functional activity of ex-vivo produced ProT-NK cells. Representative flow cytometry plots of expressions of CD107a **(A)**, interferon gamma (IFNγ) **(B)** and tumor necrosis factor alpha (TNFα) **(C)** by ProT-NK D14, ProT-NK D21 or PB-NK cells upon stimulation (+K562) or not (-K562) with K562 cells for 6 hours. PB-NK, ProT-NK D14 and ProT-NK D21 were analyzed using mass cytometry after stimulation K562 cells for 6 hours. **(D)** Heatmap (left panel) displaying the median expressions of the indicated markers in $CD56^{+}$ cells in each of the donors for all the samples analyzed. UMAP plots (right panel) showing the expression of indicated intracellular proteins in $CD56^{+}$ cells as analyzed by mass cytometry for ProT-NK D14, ProT-NK D21 and day 14 expanded PB-NK cells after stimulation for 6 hours with K562 cells at 1:2 ratio of NK to K562 cells. Merged data of different donors (N=3) for each sample are represented. (E) Gating strategy for identifying dead target (T) cells in cytotoxicity assay. Representative flow cytometry plots shown in the figure correspond to an effector-to-target ratio of 2.5:1. Total cells were identified based on forward scatter (FSC) and side scatter (SSC) characteristics. Within the total cell population, target cells were selectively stained with CellTrace Violet dye (CTV) before co-incubation with effector cells (PB-NK, ProT-NK D14, or ProT-NK D21). CTV-positive target cells were gated, and the viability of these cells was assessed by distinguishing live ($7AAD^{-}$) and dead ($7AAD^{+}$) populations. Gating for live and dead target cells was validated using control conditions: target cells alone ("T only") and target cells treated with detergent ("T + Detergent").

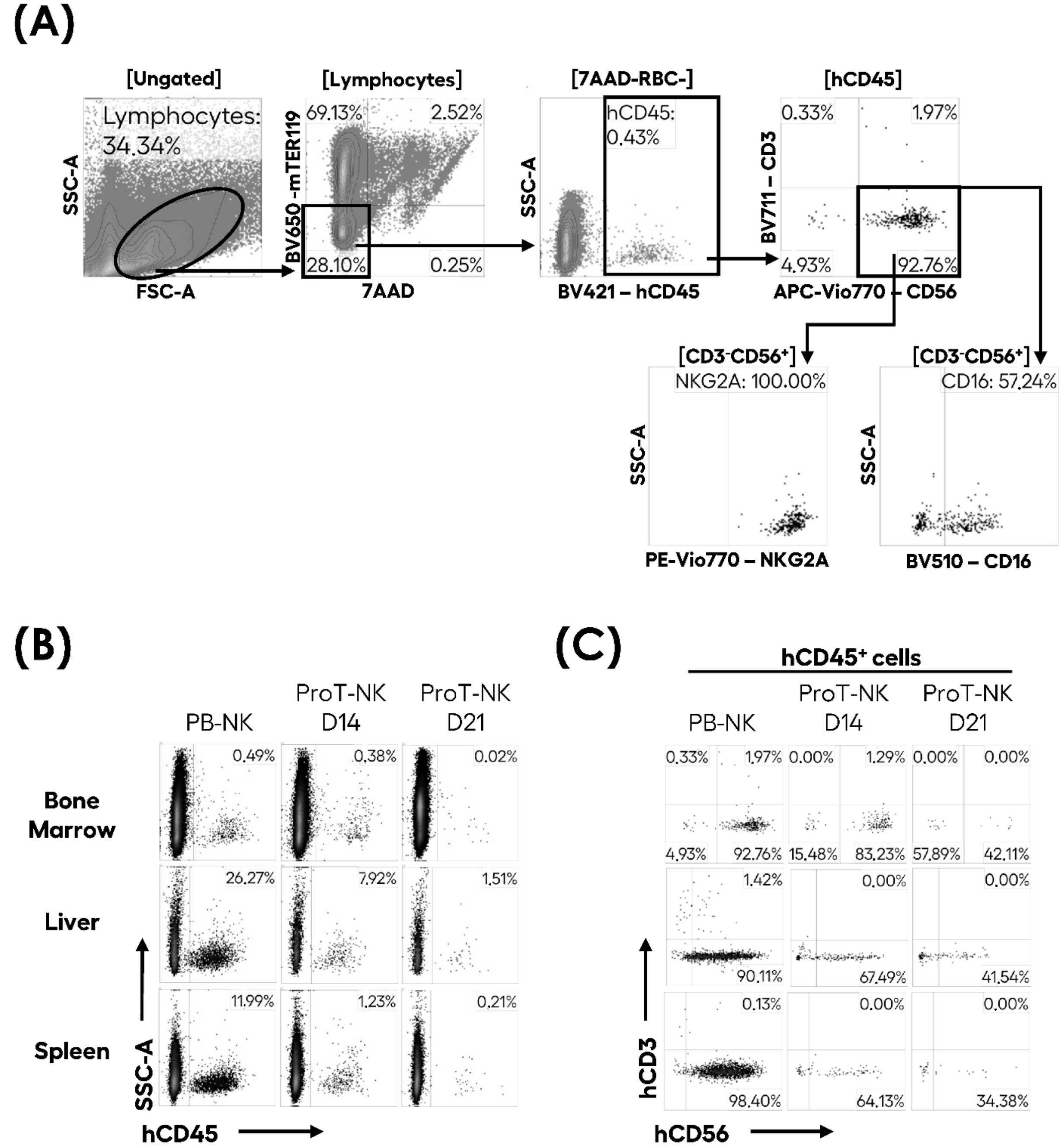

Supplementary Figure 6 – Homing potential of ProT-NK cells in NSG-Tg(hIL-15) mice. (A) Gating strategy for identifying NK cells in mouse Bone Marrow at Day 5. Lymphocytes were identified based on their forward scatter (FCS) and side scatter (SSC) characteristics. Dead cells ($7AAD^+$) and murine Red Blood Cells (RBC+, marked by mTER119) were excluded from analysis. Human cells were identified by hCD45 expression, and within this population, NK cells were defined as CD3(–) CD56(+) lymphocytes. Further gating on $CD3^-CD56^+$ NK cells was performed to analyze CD16 and NKG2A expression. Representative FC plots showing human $hCD45^+$ cells **(B)** and $CD3^-CD56^+$ population within $hCD45^+$ cells **(C)** in bone marrow, liver and spleen after 5 days of injection of 20 x 10^6 ProT-NK D14, ProT-NK D21 or PB-NK cells.
