# Supplementary material for: Feeder-cell-free system for ex vivo production of natural killer cells from cord blood hematopoietic stem and progenitor cells
Source: Front Immunol. 2025 Feb 20;16:1531736. doi: 10.3389/fimmu.2025.1531736 (PMC11883473; doi:10.3389/fimmu.2025.1531736)
Supplement: Supplementary file 2 [file Table1.pdf]

1.2 Supplementary Tables

Supplementary Table 1 - Mass cytometry antibody phenotype panel.

Label	Marker	Clone	Provider	Reference
89Y	CD45	HI30	Fluidigm	3089003B
106Cd	CD71	REA902	Miltenyi	130-124-327
110Cd	CD159c (NKG2C)	REA205	Miltenyi	130-122-278
111Cd	CCR7	G043H7	Biolegend	353237
112Cd	CD98	REA387	Miltenyi	130-122-289
114Cd	CD3	UCHT1	Biolegend	300402
114Cd	CD14	M5E2	Biolegend	301843
114Cd	CD19	HIB19	Biolegend	302247
114Cd	CD33	WM53	Biolegend	303419
116Cd	Glut1	202915	R&D Biosystems	MAB61418
141Pr	CCR6	G034E3	Fluidigm	3141003A
142Nd	CXCR1 (CD181)	8F1/CXCR1	Fluidigm	3142009B
143Nd	CD159a (NKG2A)	REA110	Miltenyi	130-122-329
144Nd	CD69	FN50	Fluidigm	3144018C
145Nd	CD16	3G8	Fluidigm	3145008B
146Nd	CD8	RPA-T8	Fluidigm	3146001B
147Sm	CXCR2 (CD182)	5E8/CXCR2	Fluidigm	3147010B
148Nd	Fas-L	REA1056	Miltenyi	130-126-491
149Sm	CD56	NCAM16.2	Fluidigm	3149021B
150Nd	KLRG1	REA261	Miltenyi	130-126-458
151Eu	CX3CR1	K0124E1	Biolegend	355702
152Sm	KIR2DS4	REA860	Miltenyi	130-122-328
153Eu	CD62-L	DREG-56	Fluidigm	3153004C
154Sm	TRAIL/TNFSF10	#75402	R&D Biosystems	MAB687
155Gd	CD 279 (PD1)	EH12.2H7	Fluidigm	3155009B
156Gd	CD195 (CCR5)	NP-6G4	Fluidigm	3156015A
158Gd	CD27	L128	Fluidigm	3158010B
159Tb	CD337 (NKp30)	Z25	Fluidigm	3159017B

160Gd	CD158a/h (KIR2DL1/S1)	REA1010	Miltenyi	130-122-345
161Dy	CD336 (NKp44)	REA1163	Miltenyi	130-126-465
162Dy	CD335 (NKp46)	BAB281	Fluidigm	3162021B
163Dy	CD183 (CXCR3)	G025H7	Fluidigm	3163004B
164Dy	CD161	191B9	Miltenyi	130-092-676
166Er	CD314 (NKG2D)	ON72	Fluidigm	3166016B
167Er	CD158e (KIR3DL1) NKB.1	DX9	Fluidigm	3167013B
168Er	CD199 (CCR9)	L053E8	Fluidigm	3168011C
169Tm	Tim-3	F38-2E2	Fluidigm	3169028B
170Er	CD122 (IL-2Rβ)	Tu27	Fluidigm	3170004B
171Yb	CD226 (DNAM-1)	DX11	Fluidigm	3171013B
172Yb	CD38	HIT2	Fluidigm	3172007C
173Yb	CD158b (KIR2DL2/L3)	DX27	Fluidigm	3173010B
174Yb	CD94	HP-3D9	Fluidigm	3174015B
175Lu	CD184 (CXCR4)	12G5	Fluidigm	3175001B
176Yb	CD57	HCD57	Fluidigm	3176019B
191Ir/193Ir	Cell-ID™ Intercalator-Ir	-	Fluidigm	201192A
195Pt	Cell-ID Cisplatin	-	Fluidigm	201064
209Bi	TIGIT	MBSA43	Fluidigm	3209013B

Supplementary Table 2 - Mass cytometry antibody function panel. Intracellular markers are labelled in italics.

Label	Marker	Clone	Provider	Reference
106Cd	CD71	REA902	Miltenyi	130-124-327
110Cd	CD159c (NKG2C)	REA205	Miltenyi	130-122-278
112Cd	CD98	REA387	Miltenyi	130-122-289
116Cd	Glut1	202915	R&D Biosystems	MAB61418
141Pr	*Granzyme A*	*REA162*	*Miltenyi*	*130-108-054*
143Nd	CD159a (NKG2A)	REA110	Miltenyi	130-122-329
144Nd	CD69	FN50	Fluidigm	3144018C
146Nd	*TNFα*	*Mab11*	*Fluidigm*	*3146010B*
148Nd	Fas-L	REA1056	Miltenyi	130-126-491
149Sm	CD56	NCAM16.2	Fluidigm	3149021B

150Nd	CD223 (LAG3)	11C3C65	Fluidigm	3165037B
151Eu	CD107a	H4A3	Fluidigm	3151002B
152Sm	KIR2DS4	REA860	Miltenyi	130-122-328
153Eu	CD62-L	DREG-56	Fluidigm	3153004C
154Sm	TRAIL/TNFSF10	#75402	R&D Biosystems	MAB687
155Gd	CD 279 (PD1)	EH12.2H7	Fluidigm	3155009B
156Gd	*Bcl-2*	*Bcl-2-100*	*Invitrogen*	*13-8800*
158Gd	CD27	L128	Fluidigm	3158010B
159Tb	CD337 (NKp30)	Z25	Fluidigm	3159017B
160Gd	CD158a/h (KIR2DL1/S1)	REA1010	Miltenyi	130-122-345
161Dy	CD336 (NKp44)	REA1163	Miltenyi	130-126-465
162Dy	CD335 (NKp46)	BAB281	Fluidigm	3162021B
163Dy	KIR2DL5	UP-R1	Miltenyi	130-096-200
164Dy	*Mip 1a/CCL3*	*REA355*	*Miltenyi*	*130-095-212*
166Er	CD314 (NKG2D)	ON72	Fluidigm	3166016B
167Er	CD158e (KIR3DL1) NKB.1	DX9	Fluidigm	3167013B
168Er	*IFNγ*	*B27*	*Fluidigm*	*3168005B*
169Tm	Tim-3	F38-2E2	Fluidigm	3169028B
170Er	CD3	UCHT1	Fluidigm	3170001B
171Yb	*Granzyme B*	*GB11*	*Fluidigm*	*3171002B*
172Yb	CD38	HIT2	Fluidigm	3172007C
173Yb	CD158b (KIR2DL2/L3)	DX27	Fluidigm	3173010B
174Yb	CD94	HP-3D9	Fluidigm	3174015B
175Lu	*Perforin*	*B-D48*	*Fluidigm*	*3175004B*
176Yb	CD57	HCD57	Fluidigm	3176019B
191Ir/193Ir	Cell-ID™ Intercalator-Ir	-	Fluidigm	201192A
195Pt	Cell-ID Cisplatin	-	Fluidigm	201064
209Bi	TIGIT	MBSA43	Fluidigm	3209013B

Supplementary Table 3 - Antibodies labelled manually using Maxpar X8 or MCP9 antibody labelling kits.

Label	Marker	Clone	Supplier	Reference
106Cd	CD71	REA902	Miltenyi	130-124-327
110Cd	CD159c (NKG2C)	REA205	Miltenyi	130-122-278
111Cd	CCR7	G043H7	Biolegend	353237
112Cd	CD98	REA387	Miltenyi	130-122-289
114Cd	CD3	UCHT1	Biolegend	300402
114Cd	CD14	M5E2	Biolegend	301843
114Cd	CD19	HIB19	Biolegend	302247
114Cd	CD33	WM53	Biolegend	303419
116Cd	Glut1	202915	R&D Biosystems	MAB61418
143Nd	CD159a (NKG2A)	REA110	Miltenyi	130-122-329
148Nd	Fas-L	REA1056	Miltenyi	130-126-491
150Nd	KLRG1	REA261	Miltenyi	130-126-458
151Eu	CX3CR1	K0124E1	Biolegend	355702
152Sm	KIR2DS4	REA860	Miltenyi	130-122-328
154Sm	TRAIL/TNFSF10	#75402	R&D Biosystems	MAB687
160Gd	CD158a/h (KIR2DL1/S1)	REA1010	Miltenyi	130-122-345
161Dy	CD336 (NKp44)	REA1163	Miltenyi	130-126-465
164Dy	CD161	191B9	Miltenyi	130-092-676
163Dy	KIR2DL5	UP-R1	Miltenyi	130-096-200
141Pr	Granzyme A	REA162	Miltenyi	130-108-054
156Gd	Bcl-2	Bcl-2-100	Invitrogen	13-8800
164Dy	Mip 1a/CCL3	REA355	Miltenyi	130-095-212
